# A genome-wide association study on hematopoietic stem cell transplantation reveals novel genomic loci associated with transplant outcomes

**DOI:** 10.3389/fimmu.2024.1280876

**Published:** 2024-02-07

**Authors:** Albert Rosenberger, Rachel E. Crossland, Ralf Dressel, Dieter Kube, Daniel Wolff, Gerald Wulf, Heike Bickeböller, Anne Dickinson, Ernst Holler

**Affiliations:** ^1^ Department of Genetic Epidemiology, University Medical Center, Georg August University Göttingen, Göttingen, Germany; ^2^ Translational & Clinical Research Institute, Faculty of Medical Science, Medical School, Newcastle University, Newcastle upon Tyne, United Kingdom; ^3^ Department of Cellular and Molecular Immunology, University Medical Center, Georg August University Göttingen, Göttingen, Germany; ^4^ Department of Internal Medicine III, University Hospital Regensburg, Regensburg, Germany; ^5^ Hematology and Medical Oncology, University Medical Center, Georg August University Göttingen, Göttingen, Germany

**Keywords:** HSCT, GvHD, GWAS, survival, competing risks

## Abstract

**Introduction:**

Data on genomic susceptibility for adverse outcomes after hematopoietic stem cell transplantation (HSCT) for recipients are scarce.

**Methods:**

We performed a genome wide association study (GWAS) to identify genes associated with survival/mortality, relapse, and severe graft-versus-host disease (sGvHD), fitting proportional hazard and subdistributional models to data of n=1,392 recipients of European ancestry from three centres.

**Results:**

The single nucleotide polymorphism (SNP) rs17154454, intronic to the neuronal growth guidant semaphorin 3C gene (*SEMA3C)*, was genome-wide significantly associated with event-free survival (p=7.0x10^-8^) and sGvHD (p=7.5x10^-8^). Further associations were detected for SNPs in the Paxillin gene (*PXN) with* death without prior relapse or sGvHD, as well as for SNPs of the Plasmacytoma Variant Translocation 1 gene *(PVT1*, a long non-coding RNA gene*)*, the Melanocortin 5 Receptor *(MC5R) gene* and the WW Domain Containing Oxidoreductase gene (*WWOX)*, all associated with the occurrence of sGvHD. Functional considerations support the observed associations.

**Discussion:**

Thus, new genes were identified, potentially influencing the outcome of HSCT.

## Introduction

Hematopoietic Stem Cell Transplantation (HSCT) was an important breakthrough in the therapy of hematologic malignancies with up to 9 HSCT per 100.000 inhabitants currently being performed in Europe each year (1, 2). More than a million HSCTs have been carried out since the first successful transplants in 1968. Transplant-related mortality (TRM) rates are decreasing from about 50% in the 1970s to as low as 10% today (2). Human leukocyte antigen (HLA) minor and major incompatibilities between HSC donors and recipients can trigger allorecognition by T-cells. Relapse and graft-versus-host disease (GvHD) are the two major reasons for transplant (Tx) failure (3). A five-year rate of relapse after HSCT can be as high as 45%-50% (2). Relapse can be treated e.g. by donor lymphocyte infusion (DLI) or novel effective pharmacological compounds, such as tyrosine kinase inhibitors (TKI), hypomethylating agents or monoclonal antibodies (4, 5).

Acute GvHD (aGvHD) results from transplanted cells attacking alloantigens on the recipient’s tissues. aGvHD affects up to 35%–45% of HLA-matched siblings and 60%–80% of partly mismatched unrelated donor transplant recipients (6). Host antigen presenting cells are activated in response to damage-associated and pathogen-associated molecular patterns (DAMPs and PAMPs) released in the course of pretransplant conditioning and induce proliferation and activation of alloreactive donor T-cells. Subsequently, these T-cells mediate local tissue injury together with soluble inflammatory agents (e.g. IFN-γ, TNF-α) (3, 6). In contrast, chronic GvHD (cGvHD) is characterized by the presence of alloreactive, dysregulatory T- and B-cells. Almost every second HSCT recipient develops cGvHD, either *de novo*, during progression or after aGvHD. Furthermore, cGvHD is a major cause of late non-relapse associated mortality following HSCT (7). Prophylaxis for GvHD was introduced as an effective preventive action. Unfortunately, the lower incidence of severe GvHD was offset by high rates of relapse in malignancy.

During the past decade, a series of genomic variants have been discussed to be associated with GvHD or other transplantation (Tx)-related outcomes (8, 9). A first genome-wide association study (GWAS) associated genotype mismatch at HLA-DPB1 (10) and rs17473423 (12p12.1, now listed as rs1137282, located in the Kirsten rat sarcoma virus (*KRAS*) gene encoding K-Ras, a part of the RAS/MAPK pathway, were associated with GvHD in a Japanese population (11). Combining GWAS on a Finnish and a Spanish cohort with gene expression studies revealed further 51 genes potentially associated with GvHD (12). However, despite plausible molecular biological explanations, replication often fails, often due to incompatibilities between patient and donor cohorts with regards to HSCT treatment regimens and patient and donor characteristics (9, 13–15).

One major challenge in searching for genetic associations with post-Tx events are the different possible clinically relevant endpoints, the competing behaviour of target events in addition to the inherent differences between transplant cohorts (16, 17). Competing post-Tx events are events which prevent the occurrence or modify the risk of each other. These include the manifestation of relapse, or GvHD and death, and need to be taken into account when assessing cause-specific hazards, cumulative incidence, event-free or overall survival (18, 19). Both previously reported GWAS used a case-control design, meaning they compare, i.e., in the simplest case, the minor allele frequency (MAF) between GvHD cases and GvHD-free controls. Such comparisons may be biased if, for example, the time since Tx of cases and controls differ, or patients are prone to both GvHD and relapse, but the considered SNP is only associated with relapse (hence in the presence of competing risks) (20).

Here, we aim to identify genomic markers, associated with any of the relevant, post-Tx key events, death, relapse and GvHD, and establish a joint GWAS using recipient DNA samples from three transplantation centres. Complex survival analysis was carried out taking competing risks into account, as previously recommended (16, 17, 19).

## Materials and methods

### Study population

This observational study is a joint analysis of samples collected in the Freeman Hospital of Newcastle (UK), the University Hospital Regensburg (Germany) and the University Medical Centre Göttingen (Germany). The patients were treated according to the respective standard of clinical protocols, between the years 2001 and 2017. All recipient and donor data were collected at the participating study centres in accordance with local standards and with the approval of the local research ethics committee. Inclusion criteria are provided in the **Supplementary Material**. All clinical data originate from the comprehensive routine documentation of the respective study centers. Patients were given the chance to follow them for at least a year.

### Sample preparation

#### Phenotype harmonization and quality control

Prior to data analysis, all phenotype data were cleaned from incorrect, inaccurate, or inconsistent entries, and harmonised between the study sites. Missing values and obvious errors have been removed or corrected after query. All dates were considered in days relative to the day of the 1^st^ Tx. All medical causes of death excluding relapse were considered as treatment-related. Survival times with unknown or clearly non-medical/non-mental causes of death were treated as censored observations. Missing values on covariates were imputed taking known information on patient, donor and treatment for each study centre into account. Notwithstanding subsequent harmonization of grading and staging (21), aGvHD and cGvHD were graded according to previously published criteria, as recorded in the study centres (22, 23). For this analysis, we jointly considered an acute GvHD of grade III or IV or an extensive chronic GvHD as severe GvHD (further noted as sGvHD), as the overall survival (OS) after onset of GvHD differs significantly between aGvHD grade I, II and III/IV (p<0.001, detail see **Supplementary Material**). We finally estimated a hazard ratios (HR) of 2.31 (95%-CI: 1.84-2.89) for sGvHD compared to aGvHD grade 1 or 2 (p<.0001), corresponding to a difference in the median survival time (after onset of GvHD) of 3100 days (~8 yrs.) versus 5664 days (~15 yrs.).

#### OncoArray genotyping and quality control

DNA samples of 1,392 patients of European ancestry were available and shipped to the Research Unit of Molecular Epidemiology of the Helmholtz Zentrum München (HGMU). Genotyping was performed using the ***Infinium*** *OncoArray 500K-V1.0* (OA) from *Illumina Inc*. in three batches between 2015 and 2017. The genotypes were subjected to strict quality control (QC) and harmonized between batches, including gender and ethnicity checks. Further details on the genotyping, quality assurance and QC are given in the **Supplementary Material**.

Overall, 499,563 markers could be genotyped. In total, 94,878 markers failed at least one of the QC-criteria. The most common reasons for exclusion were a MAF<1% or a call rate<90% (fraction of unsuccessful genotyping ≥10%) in at least one batch. After quality control (QC), 404,685 markers entered the data analysis.

We used *SNPnexus*, *dbSNP*, *dbGene*, *Ensemble*, and *Illumina* information files to assign markers to genes and other information (24–27). Pairwise linkage disequilibrium (LD) between markers was determined from *Ldlink* (28).

### Statistical analysis

#### Competing key events post Tx

We considered the course after Tx characterised by the following key events: death, or the clinical manifestation of a relapse episode, or the clinical diagnosis of sGvHD (18, 29). At least death and relapse are competing events. Other censoring events were a 2^nd^ Tx or loss-to-follow-up. Any information after the first occurrence of one of these events was ignored, to prevent therapeutic measures affecting any further post-HSCT course. If the study site documentation did not record any of the key events, or the absence of these key events was documented, we considered the respective key event as “not occurred. When describing the sample, the frequency of occurrence of a key event as the first event and the cumulative incidence function (CIF) on days 90 and 180 (Kaplan-Meier nonparametric) were reported.

#### Statistical analysis: CSH and SDH models

When evaluating time-to-event data, the hazard function is usually modelled. A hazard is understood as the instantaneous risk that an undesirable event (e.g. relapse) will occur that has not previously occurred. With competing risks, the hazards of several events (e.g. death and relapse) are correlated, because e.g. the occurrence of a relapse changes by the hazard to die. Competing risks can mutually mask the occurrence of an other event; e.g. those patients who died cannot subsequently suffer a relapse. The so-called “at-risk group” includes all study participants who are at risk for an event at time point t. How those who have experienced a competing event prior to t are treated may vary.

We considered overall survival (OS) to be the time between Tx and death, regardless of relapse or sGvHD. We defined event-free survival time (EFS) by the duration between Tx and the first occurrence of one of the key events. EFS and OS are considered as composite endpoints and therefore to be “descriptive in nature”, because key events are either ignored (OS) or undifferentiated (EFS) (16). We complemented the modelling of EFS and OS with considerations of cause-specific hazard (CSH) for sGvHD, relapse and death (as non-relapse-non-sGvHD-mortality, NRGM), as recommended (16, 19).

Cox proportional hazard regression was used as framework for the analysis. Within this we investigated cause-specific hazard ratios (HR) for all key events separately (30). In addition, we fitted Fine‐Gray’s subdistribution hazard (SDH) models, that “cure the event of interest from failure from other causes”, and allows the study of effects that interfere the incidence of the event (31–33). In cause-specific hazard (CSH) models with a key event as the outcome (e.g. death), individuals who have experienced a competing event are removed from the “risk set”. In subdistribution hazard (SDH) models, these individuals remain in the “risk set”, albeit only “partially” due to the overall survival function S(t). CSH modelling is useful for aetiological research when one wants to investigate the causal relationship between risk factors (here a SNP) and a particular outcome (e.g. sGvHD). SDH modelling is useful for prognostic research when the aim is to predict the probability of an event (e.g. sGvHD) at time t for an individual patient. The results of the two models and for all the competing key events need to be considered together in order to make a conclusive statement about a SNP. For further details see (19, 34).

Therefore, for each SNP we fitted a total of 8 models to the data (one each for OS and EFS, three each for CSH and SDH – one per key event relapse, sGvHD and NRGM). These models were fitted conditional on the study centre, adjusted for patient-, donor- or Tx-related covariates (listed in **Supplementary Table S1**) and for 3 principal components (PCs, to adjust for population stratification), as well as adjusted for PCs only (crude). To avoid over-adjustment, relevant covariates were selected in a data-driven manner using the Akaike information criterion (AIC). Modelling was performed using PROC PHREG of SAS 9.4® (35).

#### Pre-modelling of limited adequacy

Due to intensive and time-consuming modelling, we pre-selected SNPs in a fast but limited adequate modelling step, with SurvivalGWAS_SV (36). This software tool for GWAS of “time-to-event” outcomes does not allow conditioning on study centre or fitting of SDH models; and limits the number of covariates. Therefore, covariates informative for OS and EFS were combined into three scores: a “recipient score” (gender, age, previous autologous hematopoietic cell transplantation (auto-HCT), underlying condition, stage of disease, total body irradiation), a “donor/centre score” (study centre, waiting-time, patient/donor relationship, patient/donor gender-relationship), and a “Tx score” (T-cell depletion, GvHD prophylaxis, stem cell source, conditioning, reduced intensive conditioning). Details are provided in the **supplement** (**Supplementary Table S9**). Corresponding genomic inflation factors λ are given in **Supplementary Table S10**; they indicate that test results are methodologically inflated if they are greater than one.

Genome-wide significant SNPs (p ≤ 10^-7^, gwSNPs) and blocks of suggestively significant SNPs (10^-5^≤p ≤ 10^-7^; suggSNPs), as well as SNPs assigned to the same genes as gw/suggSNPs, were then reanalysed using the core statistical model described above.

#### Significance and reproducibility

The threshold for “*genome-wide significance”* was set at a level of p=10^−7^, for “*suggestive significance”* at p=10^−5^, and for “*nominal significance”* at p=0.05. We additionally demanded consistency of hazard ratios (HR; same direction and similar magnitude), respectively subdistributional hazard ratios (sHR), between subgroups and between crude vs. adjusted models. Most importantly, we demanded consistency in direction of effects (HRs) between all three study sites to demonstrate implicit replication in independent samples of different source populations. The subgroup analysis was performed in subsamples of size ≤90% of the total sample, but ≥350 (e.g. by study centre, by gender, underlying condition; in total 28 subsamples).

**Figure 1:**
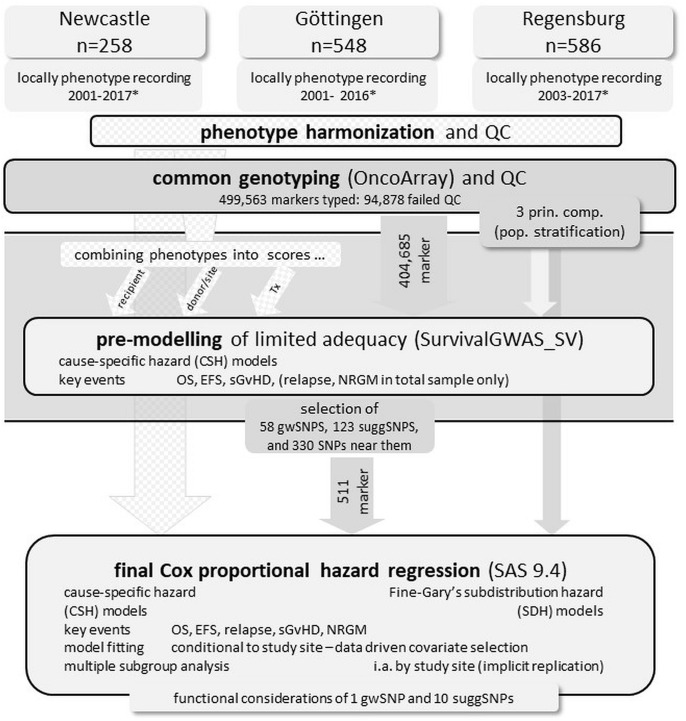
Schematic representation of the research/study strategy. * year of Tx (transplant), QC quality control, OS overall survival time, EFS event-free survival time, sGvHD severe graft-versus-host disease, NRGM non-relapse-non-sGvHD-mortality, 3 prin. comp. Three principal components of the genomic architecture to adjust for population stratification, gwSNP genome-wide significant SNPs (p ≤ 10^-7^), suggSNP suggestively significant SNPs (10^-5^≤p ≤ 10^-7^).

**Figure 1** displays a schematic of the research/study strategy.

## Results

### Sample description

The genotyped study cohort included n=1,392 recipients of European ancestry who underwent a HSCT between January 2001 and July 2017. Of these recipients, n=548 (39%) were treated in Göttingen, n=258 (19%) in Newcastle, and n=586 (42%) in Regensburg. At Tx, they were 15 to 78 years old (median 50 yrs.) and n=869 (63%) were male. The underlying disease was leukaemia in n=882 cases (63%) and lymphoma in n=473 cases (34%). In n=524 (38%) cases, the disease was in complete remission (CR), in n=340 (24%) cases in partial remission (PR), while n=189 (14%) recipients had progressive or resistant underlying disease. Most recipients (n=952, 68%) received stem cells from HLA-matched unrelated donors, with approximately 30% from HLA-matched related donors (mainly siblings) while HLA-mismatches were only present in 7% of the donors. In >90% the grafts were peripheral blood stem cells (PBSC), in 83% cases cytostatic drugs were used for conditioning, with the majority of cases (68%) receiving a reduced intensive conditioning (RIC). About 95% of recipients received GvHD prophylaxis, about 77% underwent T-cell depletion (ATG *in vivo* was applied in Regensburg und Göttingen, in Newcastle CliniMACS device® TCRαβ-Biotin system, the CliniMACS® CD34 Reagent system and ATG *in vivo* was used).

Survival (time Tx to death) was fully recorded in 277 patients. aGvHD occurred as the first key event in 98 patients (7%, CIF-90 days: 8%, CIF-180 days: 9%), cGvHD in 128 patients (9%, CIF-90 days: 3%, CIF-180 days: 18%), relapse in 113 patients (8%, CIF-90 days: 5%, CIF-180 days: 12%), and NRGM in 93 patients (8% of all patients, 33% among those whose survival is fully known, CIF-90 days: 9%, CIF-180 days: 13%). More details are tabled in the **Supplementary Material** (**Supplementary Tables S1–6**).

**Table 1** summarises some patient and donor characteristics, more details are in the **Supplementary Material**.

**Table 1:** Selected patient and donor characteristics.

Total (N=1,392)	n	%
Patient gender		
male	869	63%
female	*516*	*37%*
Underlying condition (disease)		
lymphoma	*473*	*34%*
leukaemia	*882*	*63%*
others	*5*	*<1%*
unknown	*32*	*2%*
Stage of disease at 1^st^ Tx		
Complete remission (CR)	*524*	*38%*
Partial remission (PR)	*340*	*24%*
Progressive/resistant Disease (PD/RD)	*189*	*14%*
*belonging to* CML/MPS/MPN	*74*	*5%*
*belonging to* MDS	*52*	*4%*
other	*213*	*15%*
previously auto-transplant		
yes	*149*	*11%*
Stem cell (graft) source		
PBSC	*1,246*	*90%*
Reduced Intensity Conditioning		
yes	*944*	*68%*
Total-body irradiation		
yes	*226*	*16%*
T-cells depletion		
yes	*1,065*	*77%*
GvHD Prophylaxis		
yes	*1,323*	*95%*
Conditioning		
cytostatics	*3,285*	*83%*
immunosuppressants	*11*	*<1%*
*antibodies*	*608*	*15%*
Donor lymphocyte infusion (DLI)		
yes	*133*	*10%*
Patient/Donor relationship		
HLA-matched unrelated donor	*952*	*68%*
*HLA-matched related donor*	*204*	*15%*
*siblings*	*203*	*15%*
HLA mismatch		
no	*1,275*	*92%*
Patient/Donor gender-relation		
female donor to male recipient	*232*	*17%*
Patient-Donor CMV status at 1^st^ Tx		
either or both unknown/*borderline*	47	3%
both negative	467	34%
either patient or donor positive	878	63%

### OS and EFS, genetic substructure

We observed a median EFS of 172 days (~1/2 year; 95% CI: 162-181 days) and a median OS of 789 days (~2 1/6 year, 95% CI: 662-890 days). OS and EFS rates one year after therapy were 62% and 30%, respectively; five years after therapy, 39% and 13%, respectively. Survival curves are presented in the **Supplementary Figure S1**, survival time are listed in **Supplementary Table S1**. We detected no genomic subclusters within recipients. However, the first principal component appeared to cover a minimal north-south gradient (see **Supplementary Figure S1**). Also, survival functions differ between the study centres (p_EFS_=4.6 10^-6^; see **Supplementary Figure S3**). Therefore, survival models were fitted conditional to study centres to avoid batch effects and minimize residual effects due to data collection methods, definitions of clinical endpoints, and patient management between the study sites.

### Refined analysis (candidate SNPs)

Overall, 511 SNPs (58 gwSNPS, 123 suggSNPS, listed in **Supplementary Tables S12** and **2**, and 330 SNPs near them), selected by the fast pre-modelling of limited adequacy, were subjected to a more adequate analysis (proper adjustment for selected covariables, conditioned on study centre; SDH modelling). With this we achieved one genome-wide and 10 suggestive significant signals in the total sample (see **Table 2**).

**Table 2:** Most significant findings in the total sample.

*	*marker (SNP)*	*MAF*	*location*	*gene*	*p-value*	*key event*	*model*	*type of model*
genome- wide significant								
*1*	rs17154454	3.2%	7q21.11	*SEMA3C* *(*intronic)	7.0x10^-8^	EFS	crude	EFS
suggestive significant (10^-7^)								
*2*	rs17207650	2.1%	6p21.33	ig*	1.3x10^-7^	sGvHD	adjusted	SDH
*3*	rs17142828	2.1%	16p13.3	*RBFOX1* *(*intronic)	6.6x10^-7^	EFS	crude	EFS
*4*	rs7959382	2.3%	12p11.23	*STK38L* *(*intronic)	7.7x10^-7^	sGvHD	crude	CSH
*5a*	rs1151832	12.7%	12q24.23	*PXN* *(*intronic*)*	9.9x10^-7^	NRGM	adjusted	CSH
suggestive significant (10^-6^)								
*5b*	rs4767886	12.5%	12q24.23	*PXN* *(*intronic*)*	1.3x10–^6^	NRGM	adjusted	CSH
*6*	rs9927016	5.8%	16q23.1	*WWOX* *(*intronic*)*	1.7x10^-6^	sGvHD	crude	SDH
*8*	rs34588967	1.3%	10q22.3	*POLR3A* *(coding)*	1.9x10^-6^	NRGM	crude	SDH
*9*	rs11080686	19.1%	18p11.21	*MC5R* *(3’UTR)*	2.3x10^-6^	sGvHD	adjusted	CSH
*10*	rs138553412	1.7%	7p21.1	*ABCB5* *(*intronic*)*	2.6x10^-6^	EFS	adjusted	EFS
*11*	rs17137725	2.4%	15q12	*GABRG3* *(*intronic*)*	2.6x10^-6^	relapse	adjusted	CSH

OS, overall survival; EFS, event free survival, sGvHD, severe graft-versus-host disease, NRGM, non-relapse-non-sGvHD-mortality, adjusted adjusted for patient-, donor- or Tx-related covariates and for 3 principal components, crude adjusted for 3 principal components only; all models were fit conditional for study centre, ig* intergenic between NEU1, SNHG32.

#### Genome-wide significant SNPs

**Figure 2:**
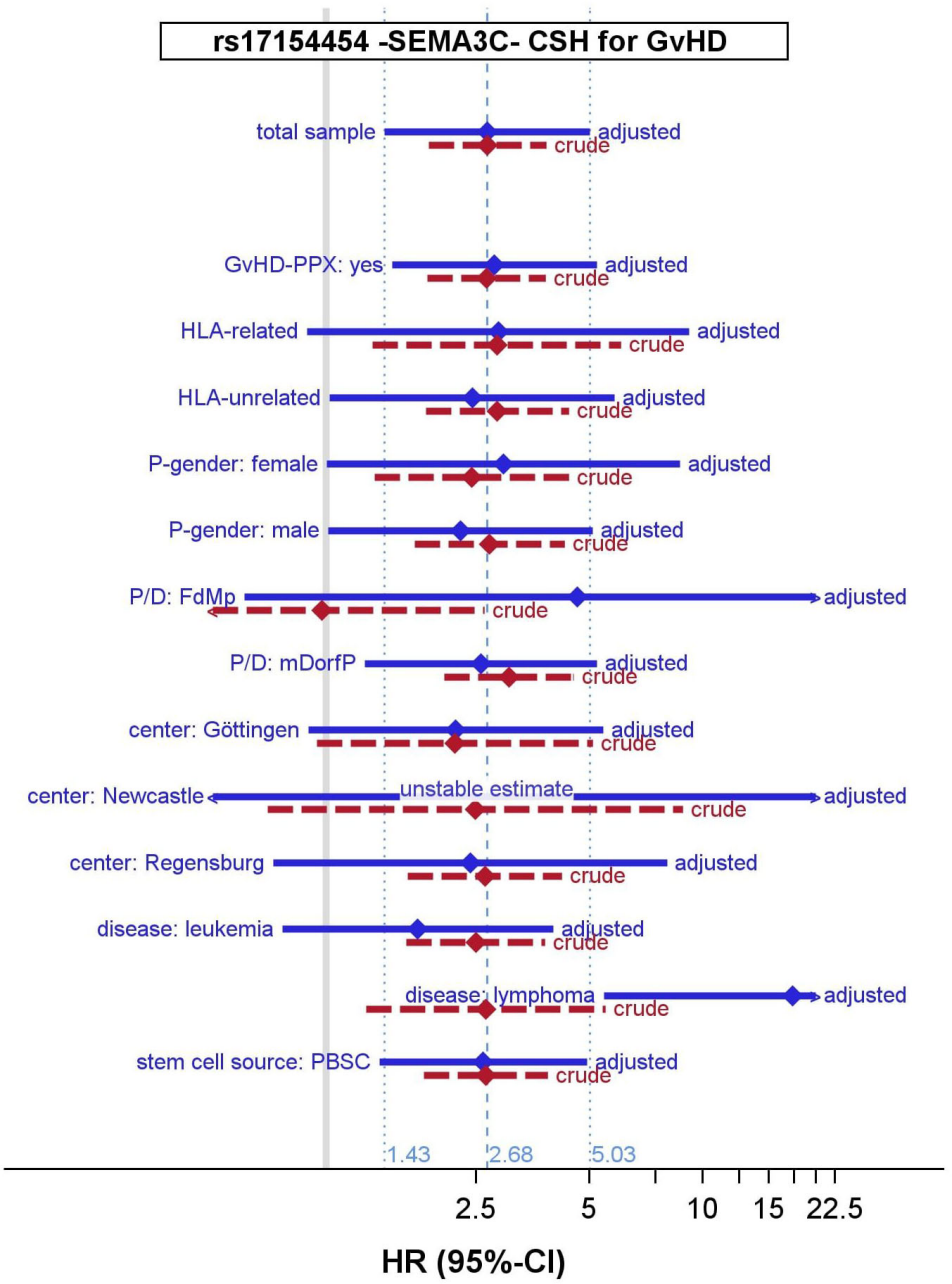
Hazard ratio (HR) estimates of rs17154454 (SEMA3C) for sGvHD by subsamples. CSH, cause-specific hazard; HR, hazard ratio, 95% CI 95% confidence interval; sGvHD, severe graft-versus-host disease; GvHD-PPX, GvHD prophylaxis; P/D, patient-to-donor relationship; FdMp, female donor-to-male recipient relationship; mDorfP, male donor-or-female recipient relationship; PBSC, peripheral blood stem cells; adjusted model adjusted for patient-, donor- or Tx-related covariates and for 3 principal components, crude adjusted for 3 principal components only.

The marker rs17154454, located at 7q21.11 intronic of *SEMA3C*, was genome-wide significantly associated with sGvHD (p=7.5x10^-8^) and to EFS (p=7x10^-8^) in the crude model (see **Table 3**). We estimated a hazard ratio of HR=2.68 (95% CI: 1.43-5.03, p=0.002) for sGvHD, adjusted for clinical features, which was comparable but less significant. The adjusted HR for EFS was slightly smaller (1.89, 95% CI: 1.22-2.93, p=0.004). Moreover, in most considered subgroups, we achieved similar HR estimates for EFS and sGvHD (see **Figure 2**). However, rs17154454 is an infrequent variant (MAF=3.2%) and hence only few recipients carry a minor allele. Thus, the sHR estimate of the SDH model was lower (adjusted: 1.40, 95% CI: 0.77-2.56, p=0.262). Therefore, the prognostic content of rs17154454 for sGvHD is limited.

**Table 3:** Hazard ratio (HR) estimates of rs17154454 (SEMA3C) by key events.

		adjusted			crude		
		p-value	(s)HR	95% CI	p-value	(s)HR	95% CI
***CSH***	**sGvHD**	0.002	2.68	1.43-5.03	7.5x10^-8^	2.68	1.87-3.84
	NRGM	0.083	2.58	0.88-7.59	0.271	1.46	0.74-2.91
	**relapse**	0.736	0.75	0.15-3.77	0.668	1.19	0.53-2.65
***EFS***	**EFS**	0.004	1.89	1.22-2.93	7.0 x10^-8^	1.86	1.48-2.34
***OS***	**OS**	0.302	1.34	0.76-2.36	0.277	1.17	0.87-1.56
***SDH***	**sGvHD**	0.262	1.40	0.77-2.56	0.0010	1.63	1.22-2.19
	NRGM	0.802	1.15	0.37-3.55	0.817	0.93	0.55-1.59
	**relapse**	0.818	1.11	0.42-2.91	0.438	0.80	0.46-1.39

CSH, cause-specific hazard model; SDH, subdistributional hazard model; (s)HR, (subdistributional) hazard ratio; OS, overall survival; EFS, event free survival, sGvHD, severe graft-versus -host disease, NRGM, non-relapse-non-sGvHD-mortality, adj. adjusted for patient-, donor- or Tx-related covariates and for 3 principal components, crude adjusted for 3 principal components only; all models were fit conditional for study centre.

#### Suggestive significant SNPs

We further achieved suggestive significant associations for markers assigned to the genes *RBFOX1*, *STK38L*, *PXN*, *WWOX*, *POLR3A*, *MC5R*, *ABCB5* and *GABRG3*; as well as for rs17207650 (a rare SNP in the intergenic region on 6p21.33/HLA region).

As in the screening step, we observed the common SNPs rs1151832 and rs4767886, located intronic of *PXN and in high LD to eachother*, as (suggestively) associated to NRGM. E.g. for rs4767886 the adjusted hazard ratio was HR=2.19 (95% CI: 1.59-3.01). In general, HR estimates were comparable between these markers, adjusted and crude modelling, and between subgroups. However, the effect was smaller and not significant in the subsample of Newcastle (e.g. adjusted: HR=1.38, 95% CI: 0.27-7.00, p=0.695). The p-values of an association to EFS or OS are <0.05 for both markers, but not to sGvHD or relapse. We also observed suggestive associations of both markers in the SDH model (e.g. rs4767886 adjusted: 1.87, 95% CI: 1.41-2.46). This together with a MAF of 12.7% indicates that *PXN* genotypes may have some prognostic value for NRGM.

We could suggestively associate the marker rs11080686, located at 19p11.21 downstream (3’UTR) of the gene *MC5R*, with the appearance of sGvHD (adjusted HR 1.54, 95% CI: 1.28-1.84, p=2.3x10^-6^). No association was seen with NRGM, relapse or OS. All hazard ratio estimates were comparable between crude and adjusted modelling, as well as between subgroups. Interestingly, the hazard ratio estimated in lymphoma patients (1.13, 95% CI: 0.81-1.57, p=0.442) was not significant and smaller than the estimate in leukemia patients (1.67, 95% CI: 1.33-2.09, p=7.9x10^-6^). We also achieved a suggestive p-value for the key event sGvHD in the SDH model (p=3.7x10-6). Together with a MAF of 19.1%, this indicates that *MC5R* genotypes may have some prognostic value for sGvHD.

For the marker rs9927016, located at 16q23.1 (spanning *FRA16D*, one of the most active common fragile sites in the human genome) within the gene *WWOX*, we observed a suggestive significant association with sGvHD (adjusted sHR=1.59, 95% CI: 1.22-1.84) in the SDH model (37). For the crude association we achieved a p=1.7x10^-6^, but adjusted for clinical features of p=0.0006 only. However, the adjusted hazard ratio estimates in the subsamples of Newcastle and Regensburg (both HR=1.69) and the crude estimate of Göttingen (HR=1.41) were consistently close.

Finally, for the marker rs17137725, located at 15q12 within the *GABRG3* gene, we observed a suggestive significant association with relapse (adjusted HR=3.80, 95% CI 2.18-6.65, p=2.6x10^-06^), but neither for sGvHD nor for NRGM. Although the direction of hazard ratio estimates is the same in all considered subgroups, there is some inhomogeneity in the magnitude between the study centres. Interestingly, larger hazard ratios were also observed within HLA-unrelated recipient/donor-pairs (HR=6.11, 95% CI 2.79-13.3) and in the subgroup “male donor or female patient” (HR=5.60, 95% CI 3.10-10.1).

The remaining findings of suggestive evidence (rs17207650 (intergenic, sGvHD); rs17142828 (*RBFOX1*, EFS/sGvHD); rs7959382 (*STK38L*, sGvHD); rs34588967 (*POLR3A*, NRGM); and rs138553412 (*ABCB5*, EFS)) belonged to rare markers and results did not prove to be robust between crude and adjusted modelling or between subgroups. They should therefore be viewed with caution (details can be seen in the **Supplementary Material**).

## Discussion

We performed GWAS on post-Tx course with sGvHD, relapse, or death as the key events. Because these events compete, it is not advisable to focus on any one of these events alone, or examine composite endpoints (like EFS) or strongly simplified endpoints (like OS). In order to understand or biologically explain an observed association of a genomic marker, one needs to know which endpoint it is associated with and which one it is not. This is not possible, if only EFS, OS or one simple event is considered. Instead, we fitted models for aetiological and prognostic research, and for all competing key events to obtain an overall view of each SNP. We did not prioritize individual SNPs, nor did we include previous findings or speculation about a SNP in our analysis.

In general, identifying genes associated with the post-Tx events remains challenging due to disease- and treatment-related confounding, competing key events, multiple tests, among others. Examination of rare markers (MAF <5%) in the post-Tx course of HSCT is difficult and prone to inexplicably low p-values.

However, we found interesting genome-wide and suggestive significant signals for several genes as described below:

The gene SEMA3C was found to be positively associated with EFS or sGvHD in the final analysis, with genome-wide significance. Many types of cancer cells express class 3 semaphorins, to inhibit tumour angiogenesis and growth. In contrast, expression of *SEMA3C* is potentiating tumour progression in several cancer types. Subsequently, *SEMA3C* displays pro- and anti-tumorgenetic characteristics (38–40). Due to its role in neurodevelopmental processes which are frequently dysregulated in cancer, inhibiting *SEMA3C* signalling is proposed as therapeutic strategy to improve cancer control (39). *SEMA3C* may contribute to drug-induced resistance of cancer cells (27, 41). Interestingly, semaphoring C has also been shown to be involved in angiogenesis, and angiogenesis has been reported as a pathogenic factor in early GvHD, and it will be of interest to see whether there is a direct interaction of semaphoring C and endothelial repair in the context of allogeneic SCT (42–46). Another semaphoring Sema4D has been linked with T cell function in the setting of GvHD, but the mechanisms may be different form our observation (47).

The gene *PXN* was the only one that appeared at least suggestively significant regarding NRGM at the screening step and in the refined analysis. Paxillin functions as a docking protein for binding signalling molecules to a specific cellular compartment. As a central protein of focal adhesion, it is a common target of many different oncoproteins and involved in numerous processes. *PXN* is considered a susceptibility gene for many types of tumours, including large B-cell lymphoma (48, 49). Upregulated *PXN* expression was linked to poor OS in acute myeloid leukaemia (50) and has been associated with prediction of relapse in chronic myeloid leukaemia (51). Therefore, PXN seems to play a role in the post-Tx course, but for which key event remains unclear.

We were able to detect three further genes that are associated with the occurrence of severe GvHD: Plasmacytoma Variant Translocation 1 (*PVT1)*, WW Domain Containing Oxidoreductase (*WWOX)* and Melanocortin 5 receptor (MC5R).

*PVT1* is considered as a candidate oncogene, and was associated e.g. with Hodgkin lymphoma (HL) (27). *PVT1* is closely located in the genome to the *MYC* gene, an universal amplifier of transcription associated with e.g. leukaemia or Burkitt lymphoma (52). They are considered “tween players among [some] haematological malignancies.” (53) Up-regulation of *PVT1*, mediated by *MYC*, is thought to confer a proliferation advantage to malignant cells, e.g. in acute myeloid leukaemia (AML), acute promyeloid leukaemia (APL), or multiple myeloma (MM) (53). *PVT1* and the circularization of the exon 2 of the *PVT1* gene (*circPVT1)*, an alternative transcript, have been reported to enhance malignant cells and hinder the immune response to the tumour during cancer progression (53). Further, two independent variants of *PVT1* (rs13255292 and rs4733601) were associated with diffuse large B-cell lymphoma (DLBCL), with genome-wide significance (54). These two markers, which were significant in our cohort, are in low LD to each other (D’ ≤ 0.357).

*WWOX* is a gene with many faces. Being located at a common fragile site (16q23.3) at the genome, it is considered a tumor suppressor gene. An increased incidence of lymphomas was observed in mouse experiments (55). However, *WWOX* is involved in several biologic functions interacting with many proteins (56). *WWOX* is involved in, inter alia, TGFβ1- and TNF-mediated cell death. *WWOX* inhibits Wnt-signaling and may control genotoxic stress-induced cell death synergistically with p53/TP53 (27, 57). *WWOX* has already been associated with severe aGvHD in a previous GWAS (p=1.75x10^−7^) (58). Defects in the gene *WWOX* are associated further with multiple types of cancer, i.a. hematopoietic malignancies (37, 59).

The protein encoded by *MC5R* is a receptor for melanocyte-stimulating and adrenocorticotropic hormones. It seems to play a role in sebum generation, but little is known about its involvement of tumorigenesis (27). In a small mouse experiment, *MC5R* was found as the only melanocortin receptor mediating the α-melanocyte-stimulating hormone (α-MSH) that promotes myelopoiesis, which in turn has been associated with GvHD in other animal models (60, 61).

Two other markers intronic of *KCNS3* and *LINC02570* were of genome-wide significance with respect to OS, but only at the screening step. The lack of a reasonable functional explanation and the lack of significance in the refined analysis limit the evidence for this finding.

Finally, the gene *GABRG3* appeared suggestively significant related to relapse. The protein (a gamma-aminobutyric acid receptor) encoded by *GABRG3* is the major inhibitory neurotransmitter in the mammalian brain (27). It contains the benzodiazepine binding site. Benzodiazepine use during HSCT was associated with adverse effects, as is the case in non-transplant patients (62, 63).. The GABAergic system is involved in immune cell functions, inflammatory conditions and diseases in peripheral tissues (64, 65). It was found that GABA protects human islet cells against the deleterious effects of immunosuppressive drugs and exerts immunoinhibitory effects (66).

Although we used all available data collected over a long period within three transplantation centres, the sample size is rather small for a GWAS considering three competing key events, due to the problem of multiple testing. We found it necessary to fit many and rather complex models in order to adequately apply Cox’s proportional hazard models to investigate the impact of individual SNPs on the course on the post-transplant outcome. While this is time-consuming and computationally expensive, it is the only way to separately assess their impact on key events while examine them in their entirety. Appropriate adjustment for clinical covariates appears to be another key to reliable estimation results. Thus, we were unable to run a single genome-wide analysis in suitable time with feasible use of computer resources. It is likely, that potentially associated markers were not selected during the screening step. We also restricted our investigation to recipient genotypes. A further differentiation between GvHD of different stages would be desirable, but only advisable in larger samples. Although we conducted some subgroup analyses, we did not fully investigate the interaction with clinical or environmental factors.

The observed suggestive association of a GABRG3 marker and relapse could be due to the administration of benzodiazepine during HSCT. Since the use of narcotic analgesics and psychotropic drugs was not considered as clinical covariate (data not available), the association, although unlikely, may be caused by residual confounding. If this is the case, it justifies the need for an appropriate adjustment when studying associations between gene and HST outcomes.

## Conclusion

We found the recipient SNP rs17154454 of *SEMA3C* to be associated with EFS and severe GvHD. In addition recipient SNPs in the genes *PXN*, *PVT1*, *MC5R* and *WWOX* potentially influence the outcome of HSCT. All discovered genes are directly or indirectly involved in molecular pathways that could affect GvHD, relapse or other key events after HSCT. However, identifying genes associated with the post-Tx events remains challenging due to disease- and treatment-related confounders, competing key events, and multiple tests.

## Data availability statement

The datasets presented in this article are not readily available because of legal and privacy restrictions. Requests to access the datasets should be directed to arosenb@gwdg.de.

## Ethics statement

The studies involving humans were approved by the Ethical Review Committee of the University of Regensburg (approval no 02-220), the Ethical Review Committee of the University Medical Center Göttingen (approval no 6/7/10) and the Newcastle and North Tyneside Research Ethics Committee (REC Red: 14/NE/1136 and 07/H0906/131). The studies were conducted in accordance with the local legislation and institutional requirements. Written informed consent for participation in this study was provided by the participants’ legal guardians/next of kin.

## Author contributions

AR: Formal analysis, Methodology, Software, Writing – original draft. RC: Writing – review & editing, Resources. RD: Writing – review & editing. DK: Resources, Writing – review & editing. DW: Resources, Writing – review & editing. GW: Resources, Writing – review & editing. HB: Funding acquisition, Writing – review & editing, Supervision. AD: Resources, Writing – review & editing, Conceptualization, Funding acquisition. EH: Funding acquisition, Writing – review & editing, Conceptualization, Resources.
