# Supplementary material for: A genome-wide association study on hematopoietic stem cell transplantation reveals novel genomic loci associated with transplant outcomes
Source: Front Immunol. 2024 Feb 7;15:1280876. doi: 10.3389/fimmu.2024.1280876 (PMC10879589; doi:10.3389/fimmu.2024.1280876)
Supplement: Supplementary file 1 [file DataSheet_1.docx]

Supplement 1

to: A genome-wide association study on hematopoietic stem cell transplantation reveals novel genomic loci associated with GvHD and Relapse
Albert Rosenberger et al.

# Inclusion criteria (patient/recipient)

Inclusion criteria for genotyping:

1. Age ≥15 at time of HSCT
2. HSCT after 2000; i.e. starting 2001
3. Clinical data are available, especially the primary outcome aGvHD (Grade 0,...,4) is known
4. Sufficient DNA quality for GWAS chips is present: 15µl (>=60 ng/µl)
5. First allo transplant (i.e. no multiple transplants)
6. No previous GWAS genotyping

# Phenotype definition and harmonization

Waiting time was defined as the period between diagnosis and Tx.

The patient-donor relationship was classified as a familial/non-familial/sibling relationship, additionally as HLA-matched or mismatched, or otherwise. Infection with cytomegalovirus (CMV) at Tx was considered as “either patient or donor CMV positive”, “both CMV negative” or “either patient or donor unknown”. The underlying condition reported (base disease) was classified as lymphoma, leukaemia, other or unknown, with further subclassification (listed in Supplementary S-Table 5). The stage of disease at Tx was considered as complete (CR) or partial remission (PR), progressive or resistant disease (PD/RD), belonging to chronic myeloid leukaemia / myeloproliferative syndromes / myeloproliferative neoplasm (CML/MPS/MPN), belonging to myelodysplastic syndromes (MDS) or unknown; regardless of previous remissions or relapses (listed in Supplementary S-Table 5). Recorded conditioning regimens were grouped into antibodies, cytostatic (topoisomerase inhibitor, alkylates, antimetabolite, other cytostatic), immunosuppressant, or other (including standard and unknown).

# Genotyping

The ***Infinium*** *OncoArray 500K-V1.0* (OA) consists of a genome-wide backbone of 230,000 SNPs marking the most common genetic variants, complemented by rare variants of pharmacogenetic- and cancer-related markers. (1)

Genotyping was carried out with the following characteristics:

- Software used: Genome Studio Version 2011.1 (Illumina)
- Genotyping modules: 1.9.4.
- Subjects with "poor" quality of DNA were analyzed and possibly repeated
- Cross-sample checks to control samples from HapMap and duplicate genotyping
- Used manifest File: OncoArray-500K-C.bpm
- Used cluster file: onco_v2c.egt (cluster file of OncoArray Networks)

Quality Control per marker

The following QC criteria per marker were tested:

1. Call rate = 0% (complete loss)
2. Arrays with complete loss or reduced call rate
3. Call rate <90%
4. MAF = 0% (monomorphic markers)
5. MAF <1% (rare alleles)
6. HWE among all (p <1x10^-12^)
7. Significant call rate differences between men and women (p <1x10^-12^)
8. Comparison of MAF with a reference population

Quality Control per sample (person)

The following QC criteria per sample were tested:

1. Missing values (missingness) per person (call rate <95%)
2. Accumulation at low call rate per chromosome
3. Gender check
4. Inbreeding and heterozygosity per person
5. Genomic relationships (cryptic and expected relatedness)
6. Genomic stratification
7. Clusters of samples with respect to missing value patterns
8. Pairwise comparison regarding patterns of missing values
9. European ancestry

Merging genotyping batches

The data sets of the three genotyping batches (2015, 2016 and 2017) were harmonized and merged by:

1. unifying the marker labeling and position;
2. issuing new unique IDs if the same person was genotyped multiple times between batches;
3. completion of the allele coding, in the case markers are monomorphic in one batch;
4. defining the allele coding for completely missing markers in one batch;
5. harmonizing the allele coding to the reference allele coding for European ancestry provided by Illumina; and
6. checking the similarity of the MAF per marker between batches and a reference for European ancestry provided by Illumina.

Genetic Substructure

S-Figure 1 Genomic structure of the study sample


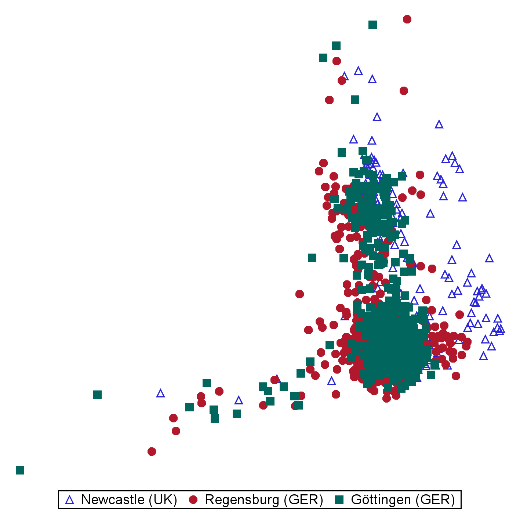

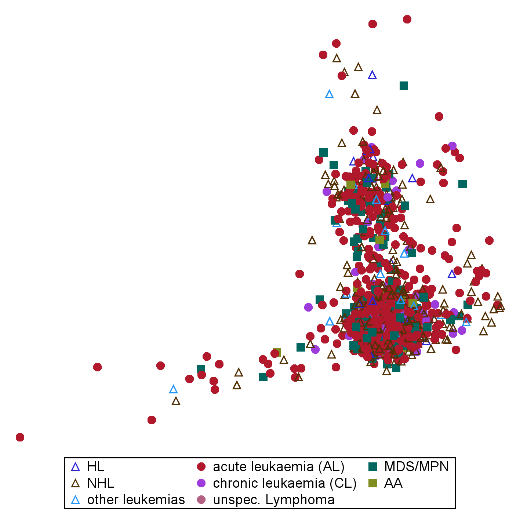


First vs. second principal component representing the genome-wide similarities;
left: grouped by study center; right: grouped by underlying disease.

# Patient and donor characteristics

As expected in three independently operating centres, most patient and donor characteristics were distributed differently. Homogeneity across study samples was seen solely for gender and the recipients-donors relationship. Therefore appropriate adjustment of relevant covariates was required.

S-Table 1 Patient characteristics

| Patient characteristics | | all | | Göttingen | | Newcastle | | Regensburg | |
| --- | --- | --- | --- | --- | --- | --- | --- | --- | --- |
| Total | | N=1,392 |  | N=548 | (39%) | N=258 | (19%) | N=586 | (42%) |
|  | |  | |  | |  | |  | |
|  | | n | % | n | % | n | % | n | % |
| **Single/multiple-donor Tx** | |  |  |  |  |  |  |  |  |
| single donor | | *1,387* | *100%* | *548* | *100%* | *258* | *100%* | *581* | *100%* |
| multiple donors | | *5* | *<1%* |  |  |  |  | *5* | *<1%* |
| **Patient ethnicity** | |  |  |  |  |  |  |  |  |
| European ancestry (assumed) | | *1,385* | *100%* | *541* | *100%* | *258* | *100%* | *586* | *100%* |
| **Patient gender** (p=0.3989) | |  |  |  |  |  |  |  |  |
| male | | 869 | 63% | 332 | 61% | 171 | 66% | 366 | 62% |
| female | | *516* | *37%* | *209* | *39%* | *87* | *34%* | *220* | *38%* |
| **Underlying condition (disease)** (p<0.0001) | |  |  |  |  |  |  |  |  |
| lymphoma | | 473 | 34% | 209 | 38% | 96 | 37% | 168 | 29% |
| aplastic anaemia (AA) | | *22* | *2%* | *10* | *2%* | *2* | *<1%* | *10* | *2%* |
| non-Hodgkin lymphoma (NHL) | | *407* | *29%* | *187* | *34%* | *80* | *31%* | *140* | *24%* |
| Hodgkin lymphoma (HL) | | *34* | *2%* | *12* | *2%* | *14* | *5%* | *8* | *1%* |
| unspecific lymphoma | | *10* | *<1%* | *--* | *--* | *--* | *--* | *10* | *2%* |
| leukaemia | | 882 | 63% | 334 | 61% | 143 | 55% | 405 | 69% |
| acute leukaemia (AL) | | *638* | *46%* | *260* | *47%* | *102* | *40%* | *276* | *47%* |
| other leukaemia | | *27* | *2%* | *10* | *2%* | *--* | *--* | *17* | *3%* |
| chronic leukaemia (CL) | | *60* | *4%* | *13* | *2%* | *18* | *7%* | *29* | *5%* |
| MDS/MPN | | *157* | *11%* | *51* | *9%* | *23* | *9%* | *83* | *14%* |
| Others | | 5 | <1% | 2 | <1% | 2 | <1% | 1 | <1% |
| Unknown | | 32 | 2% | 3 | <1% | 17 | 7% | 12 | 2% |
| **Stage of disease at 1^st^ Tx** (p<0.0001) | |  |  |  |  |  |  |  |  |
| Partial remission (PR) | | *340* | *24%* | *136* | *25%* | *27* | *10%* | *177* | *30%* |
| belonging to CML/MPS/MPN | | *74* | *5%* | *22* | *4%* | *15* | *6%* | *37* | *6%* |
| Complete remission (CR) | | *524* | *38%* | *175* | *32%* | *172* | *67%* | *177* | *30%* |
| belonging to myelodysplastic syndromes (MDS) | | *52* | *4%* | *19* | *3%* | *4* | *2%* | *29* | *5%* |
| Progressive/resistant Disease (PD/RD) | | *189* | *14%* | *133* | *24%* | *5* | *2%* | *51* | *9%* |
| Increase in disease burden or new sites of disease (Progression) | | *63* | *5%* | *18* | *3%* | *5* | *2%* | *40* | *7%* |
| Unknown | | *147* | *11%* | *45* | *8%* | *30* | *12%* | *72* | *12%* |
| untreated | | *3* | *<1%* | *--* | *--* | *--* | *--* | *3* | *<1%* |
| **Overall survival time [censoring]** | |  |  |  |  |  |  |  |  |
| not determinable | | *18* | *1%* | *4* | *<1%* | *14* | *5%* | *--* | *--* |
| death | | *639* | *46%* | *277* | *51%* | *101* | *39%* | *261* | *45%* |
| censored | | *673* | *48%* | *254* | *46%* | *128* | *50%* | *291* | *50%* |
| sGvHD | | *51* | *4%* | *6* | *1%* | *15* | *6%* | *30* | *5%* |
| relapse | | *7* | *<1%* | *7* | *1%* | *--* | *--* | *--* | *--* |
| 2^nd^ transplant | | *4* | *<1%* | *--* | *--* | *--* | *--* | *4* | *<1%* |
| **Has the patient previously had an auto-transplant** (p=0.3397) | |  |  |  |  |  |  |  |  |
| unknown | | *698* | *50%* | *548* | *100%* | *20* | *8%* | *130* | *22%* |
| no | | *545* | *39%* | *--* | *--* | *182* | *71%* | *363* | *62%* |
| yes | | *149* | *11%* | *--* | *--* | *56* | *22%* | *93* | *16%* |
| **Stem cell (graft) source** (p<0.0001) | |  |  |  |  |  |  |  |  |
| BM | | *114* | *8%* | *23* | *4%* | *34* | *13%* | *57* | *10%* |
| CB | | *6* | *<1%* | *--* | *--* | *1* | *<1%* | *5* | *<1%* |
| PBSC | | *1,246* | *90%* | *521* | *95%* | *204* | *79%* | *521* | *89%* |
| PBSC+BM | | *3* | *<1%* | *--* | *--* | *--* | *--* | *3* | *<1%* |
| unknown | | *23* | *2%* | *4* | *<1%* | *19* | *7%* | *--* | *--* |
| **Reduced Intensity Conditioning** (p<0.0001) | |  |  |  |  |  |  |  |  |
| unknown | | *20* | *1%* | *5* | *<1%* | *--* | *--* | *15* | *3%* |
| no | | *428* | *31%* | *248* | *45%* | *72* | *28%* | *108* | *18%* |
| yes | | *944* | *68%* | *295* | *54%* | *186* | *72%* | *463* | *79%* |
| **Total-body irradiation** (p<0.0001) | |  |  |  |  |  |  |  |  |
| unknown | | *4* | *<1%* | *4* | *<1%* | *--* | *--* | *--* | *--* |
| no | | *1,162* | *83%* | *498* | *91%* | *216* | *84%* | *448* | *76%* |
| yes | | *226* | *16%* | *46* | *8%* | *42* | *16%* | *138* | *24%* |
| **T-cells depletion** (p<0.0001) | |  |  |  |  |  |  |  |  |
| unknown | | *25* | *2%* | *4* | *<1%* | *13* | *5%* | *8* | *1%* |
| no | | *302* | *22%* | *117* | *21%* | *36* | *14%* | *149* | *25%* |
| yes | | *1,065* | *77%* | *427* | *78%* | *209* | *81%* | *429* | *73%* |
| **GvHD Prophylaxis** (p<0.0001) | |  |  |  |  |  |  |  |  |
| unknown | | *51* | *4%* | *1* | *<1%* | *49* | *19%* | *1* | *<1%* |
| no | | *18* | *1%* | *4* | *<1%* | *--* | *--* | *14* | *2%* |
| yes | | *1,323* | *95%* | *543* | *99%* | *209* | *81%* | *571* | *97%* |
| **Conditioning** (p<0.0001) | |  |  |  |  |  |  |  |  |
| cytostatics | | *3,285* | *83%* | *1254* | *74%* | *450* | *67%* | *1581* | *98%* |
| topoisomerase inhibitors | | *18* | *<1%* | *10* | *<1%* | *2* | *<1%* | *6* | *<1%* |
| alkylates | | *1,840* | *46%* | *766* | *45%* | *226* | *34%* | *848* | *53%* |
| antimetabolites | | *1,299* | *33%* | *435* | *26%* | *205* | *30%* | *659* | *41%* |
| other cytostatics | | *128* | *3%* | *43* | *3%* | *17* | *3%* | *68* | *4%* |
| immunosuppressants | | *11* | *<1%* | *--* | *--* | *11* | *2%* | *--* | *--* |
| antibodies | | *608* | *15%* | *426* | *25%* | *170* | *25%* | *12* | *<1%* |
| Other | | *87* | *2%* | *18* | *1%* | *52* | *7%* | *15* | *<1%* |
| Unknown | | *67* | *2%* | *18* | *1%* | *34* | *5%* | *15* | *<1%* |
| **Any relapse post Tx [yes/no]** | |  |  |  |  |  |  |  |  |
| unknown | | *777* | *56%* | *382* | *70%* | *148* | *57%* | *247* | *42%* |
| no | | *258* | *19%* | *--* | *--* | *36* | *14%* | *222* | *38%* |
| yes | | *357* | *26%* | *166* | *30%* | *74* | *29%* | *117* | *20%* |
| **Patient CMV status at 1^st^ Tx** (p=0.0726) | |  |  |  |  |  |  |  |  |
| unknown | | *41* | *2%* | *21* | *3%* | *19* | *7%* | *1* | *<1%* |
| negative | | *647* | *46%* | *241* | *44%* | *115* | *45%* | *291* | *50%* |
| positive | | *704* | *51%* | *286* | *52%* | *124* | *48%* | *294* | *50%* |
| **Donor lymphocyte infusion (DLI)** | |  |  |  |  |  |  |  |  |
| unknown | | *803* | *58%* | *11* | *2%* | *258* | *100%* | *534* | *91%* |
| no | | *456* | *33%* | *456* | *83%* | *--* | *--* | *--* | *--* |
| yes | | *133* | *10%* | *81* | *15%* | *--* | *--* | *52* | *9%* |
|  | | *n* | *mean* | *median* | | *min* | | *max* | |
| **age at 1^st^ Tx** | *Total* | 1,373 | 50 | 52 | | 15 | | 78 | |
| (p<0.0001) | *Göttingen* | 541 | 53 | 54 | | 17 | | 78 | |
|  | *Newcastle* | 246 | 46 | 47 | | 19 | | 70 | |
|  | *Regensburg* | 586 | 50 | 52 | | 15 | | 70 | |
| **waiting time [days]** | *Total* | 1,355 | -747 | -323 | | -10757 | | -34 | |
| **(diag.-1.st Tx))** | *Göttingen* | 532 | -650 | -312 | | -7,919 | | -58 | |
| (p<0.0001) | *Newcastle* | 238 | -1,005 | -428 | | -7,700 | | -97 | |
|  | *Regensburg* | 585 | -729 | -307 | | -10757 | | -34 | |
| **transplant year** | *Total* | 1,385 | 2011 | 2012 | | 2001 | | 2017 | |
| (p<0.0001) | *Göttingen* | 541 | 2012 | 2013 | | 2001 | | 2017 | |
|  | *Newcastle* | 258 | 2011 | 2012 | | 2001 | | 2016 | |
|  | *Regensburg* | 586 | 2010 | 2010 | | 2001 | | 2017 | |
| **date of 1^st^ Tx** | *Total* |  |  |  | | 23/01/2001 | | 11/07/2017 | |
|  | *Göttingen* |  |  |  | | 05/02/2001 | | 16/11/2016 | |
|  | *Newcastle* |  |  |  | | 23/01/2001 | | 11/07/2017 | |
|  | *Regensburg* |  |  |  | | 05/03/2003 | | 24/05/2017 | |

CML chromic myeloid leukaemia, MPS myeloproliferative syndromes, MPN myeloproliferative neoplasm, MDS myelodysplastic syndromes, 1st Tx first stem cell transplantation, CMV Cytomegalovirus, BM bone marrow, CB cord blood, PBSC peripheral blood stem cells

S-Table 2 Patient-Donor characteristics

|  | | all | | Göttingen | | Newcastle | | Regensburg | |
| --- | --- | --- | --- | --- | --- | --- | --- | --- | --- |
| Total | | N=1,392 |  | N=548 | (39%) | N=258 | (19%) | N=586 | (42%) |
|  | |  | |  | |  | |  | |
|  | | n | % | n | % | n | % | n | % |
| **Patient/Donor relationship** (p<0.0001) | |  |  |  |  |  |  |  |  |
| HLA-matched unrelated donor (MUD) | | 952 | 68% | 387 | 71% | 150 | 58% | 415 | 71% |
| HLA-matched related donor* (MRD) | | 204 | 15% | 147 | 27% | 25 | 10% | 32 | 5% |
| Siblings | | 203 | 15% | -- | -- | 68 | 26% | 135 | 23% |
| other relationship | | 22 | 2% | 10 | 2% | 8 | 3% | 4 | <1% |
| unknown | | 11 | <1% | 4 | <1% | 7 | 3% | -- | -- |
| **HLA mismatch** (p<0.0001) | |  |  |  |  |  |  |  |  |
| unknown | | 24 | 2% | 4 | <1% | 16 | 6% | 4 | <1% |
| no | | 1,275 | 92% | 452 | 82% | 242 | 94% | 581 | 99% |
| yes | | 93 | 7% | 92 | 17% | -- | -- | 1 | <1% |
| **Patient/Donor gender-relation** (p=0.8142**)** | |  |  |  |  |  |  |  |  |
| male donor and/or female patient | | 1,160 | 83% | 461 | 84% | 214 | 83% | 485 | 83% |
| female donor to male recipient | | 232 | 17% | 87 | 16% | 44 | 17% | 101 | 17% |
| **Patient-Donor CMV status at 1^st^ Tx** (p=0.0081) | |  |  |  |  |  |  |  |  |
| either or both unknown | | 47 | 3% | 27 | 5% | 20 | 8% | -- | -- |
| both negative | | 467 | 34% | 155 | 28% | 87 | 34% | 225 | 38% |
| either patient or donor positive | | 878 | 63% | 366 | 67% | 151 | 59% | 361 | 62% |
|  | | *n* | *mean* | *median* | | *min* | | *max* | |
| **Donor age at 1^st^ Tx** | *Total* | 1,023 | 40 | 40 | | 0 | | 71 | |
| (p=0.0448) | *Göttingen* | 337 | 41 | 41 | | 15 | | 71 | |
| **Donor age at 1^st^ Tx** | *Newcastle* | 242 | 38 | 38 | | 0 | | 68 | |
| (p=0.0448) | *Regensburg* | *444* | *40* | *40* | | *5* | | *70* | |

*HLA-matched related donor other than siblings

S-Table 3 Donor characteristics

|  | all | | Göttingen | | Newcastle | | Regensburg | |
| --- | --- | --- | --- | --- | --- | --- | --- | --- |
| Total | N=1,392 |  | N=548 | (39%) | N=258 | (19%) | N=586 | (42%) |
|  |  | |  | |  | |  | |
|  | n | % | n | % | n | % | n | % |
| **Donor-1 ethnicity** |  |  |  |  |  |  |  |  |
| European ancestry (assumed) | *1,380* | *99%* | *548* | *100%* | *258* | *100%* | *574* | *98%* |
| unknown | *12* | *<1%* | *--* | *--* | *--* | *--* | *12* | *2%* |
| **Donor-1 gender** (p=0.1429) |  |  |  |  |  |  |  |  |
| unknown | *26* | *2%* | *22* | *4%* | *4* | *2%* | *--* | *--* |
| male | *939* | *67%* | *378* | *69%* | *169* | *66%* | *392* | *67%* |
| female | *427* | *31%* | *148* | *27%* | *85* | *33%* | *194* | *33%* |
| **Donor CMV status at 1^st^ Tx** (p=0.0227) |  |  |  |  |  |  |  |  |
| unknown | *50* | *4%* | *30* | *5%* | *20* | *8%* | *--* | *--* |
| negative | *717* | *52%* | *260* | *47%* | *119* | *46%* | *338* | *58%* |
| positive | *625* | *45%* | *258* | *47%* | *119* | *46%* | *248* | *42%* |

S-Table 4 Grade of GvHD

|  | | Total | | GvHD onset grade | | | | | | GvHD max grade | | | | | |
| --- | --- | --- | --- | --- | --- | --- | --- | --- | --- | --- | --- | --- | --- | --- | --- |
|  |  | N | % | unknown | 0 | 1 | 2 | 3 | 4 | unknown | 0 | 1 | 2 | 3 | 4 |
| **aGvHD** | *unknown* | 316 | 23% | 316 | -- | -- | -- | -- | -- | 316 | -- | -- | -- | -- | -- |
|  | *no* | 357 | 26% | 357 | -- | -- | -- | -- | -- | 357 | -- | -- | -- | -- | -- |
|  | *yes* | 719 | 52% | 53 | -- | 266 | 240 | 102 | 58 | 420 | -- | 98 | 105 | 61 | 35 |
| (p<0.0001) | *Göttingen* |  |  |  |  | *90* | *79* | *60* | *33* |  |  |  |  |  |  |
|  | *Newcastle* |  |  |  |  | *49* | *43* | *4* | *1* |  |  |  |  |  |  |
|  | *Regensburg* |  |  |  |  | *127* | *118* | *38* | *24* |  |  |  |  |  |  |
| **cGvHD** | *unknown* | 373 | 27% | 373 | -- | -- | -- | -- | -- | 373 | -- | -- | -- | -- | -- |
|  | *no* | 406 | 29% | 406 | -- | -- | -- | -- | -- | 406 | -- | -- | -- | -- | -- |
|  | *yes* | 613 | 44% | 26 | 55 | 347 | 137 | 48 | -- | 439 | 1 | 31 | 70 | 72 | -- |
| (p<0.0001) | *Göttingen* |  |  |  |  | *78* | *46* | *6* |  |  |  |  |  |  |  |
|  | *Newcastle* |  |  |  |  | *120* | *--* | *--* |  |  |  |  |  |  |  |
|  | *Regensburg* |  |  |  |  | *149* | *91* | *42* |  |  |  |  |  |  |  |

# Phenotype Classification

S-Table 5 Classification of the underlying condition reported (base disease)

| Level 1 classification | Level 2 classification | Level 3 classification | underlying condition record as … |
| --- | --- | --- | --- |
| Lymphoma | **AA** | **AA** | aplastic anaemia (AA) |
|  |  |  | bone marrow failure syndromes (BMFS) |
|  |  |  | Severe Aplastic Anaemia (sAA) |
|  | **NHL** | **T-cell NHL** | Angioimmunoblastic lymphadenopathy (AILD) |
|  |  |  | Angioimmunoblastic T-cell lymphoma (AILT) |
|  |  |  | Myelofibrose (MF) |
|  |  |  | Peripheral T-cell Lymphoma (PTCL) |
|  |  |  | Peripheral T-cell Lymphoma Not Otherwise Specified(PTCL-NOS) |
|  |  |  | T-lymphoblastic Lymphoma (T-LBL) |
|  |  |  | T-cell non-Hodgkin lymphoma (T-NHL) |
|  |  |  | T-cell prolymphocytic leukaemia (T-PLL) |
|  |  |  | T-cell lymphoma |
|  |  | **B-cell NHL** | B-cell chronic lymphocytic leukaemia (B-CLL) |
|  |  |  | B-cell non-Hodgkin lymphoma (B-NHL) |
|  |  |  | B-cell lymphoma (BCL) |
|  |  |  | Burkitt lymphoma |
|  |  |  | chronic lymphocytic leukaemia (CLL) |
|  |  |  | Diffuse large B-cell lymphoma (DLBCL) |
|  |  |  | Mantle cell lymphoma (MCL) |
|  |  |  | Morbus Waldenström |
|  |  | **Plasma cell NHL** | Multiple myeloma (MM) |
|  |  |  | Plasma cell leukaemia (PCL) |
|  |  |  | Plasma cell neoplasm (PCN) |
|  |  | **unspecific NHL** | non-Hodgkin lymphoma (NHL) |
|  | **HL** | **specific HL** | Cutaneous follicle centre lymphoma (FCL) |
|  |  | **unspecific. HL** | Hodgkin lymphoma (HL) |
|  | **unspec. Lymphoma** | **unspecific. Lymphoma** | unspecified Lymphoma |
| Leukaemia | **acute leukaemia (AL)** | **unspecific. AML,ALL** | acute lymphocytic leukaemia (ALL) |
|  |  |  | Acute myeloid leukaemia (AML) |
|  |  |  | Blastic plasmacytoid dendritic cell neoplasm (BPDCN) |
|  |  |  | secondary acute myeloid leukaemia (sAML) |
|  |  | **B-cell AML,ALL** | B-cell acute lymphocytic leukaemia (B-ALL) |
|  |  |  | Common acute lymphocytic leukaemia (cALL) |
|  |  | **T-cell AML,ALL** | T-cell acute lymphocytic leukaemia (T-ALL) |
|  |  |  | T-cell acute myeloid leukaemia (T-AML) |
|  | **other leukaemia** | **Biphenotypic Leukaemia (BAL)** | Biphenotypic Leukaemia (BAL) |
|  |  | **MPN (not CML)** | Primäre Myelofibrose (PMF) |
|  |  |  | Polycythaemia vera (PV) |
|  | **chronic leukaemia (CL)** | **CML** | chronic myelogenous leukaemia (CML) |
|  | **MDS/MPN** | **MDS/MPN** | Chronic myelomonocytic leukaemia (CMML) |
|  |  |  | Myelodysplastic syndromes (MDS) |
|  |  |  | Myeloproliferative neoplasms (MPN) |
|  |  |  | Myeloproliferative Syndrome (MPS) |
|  |  |  | Refractory anaemia with excess blasts-1 (RAEB-1) |
|  |  |  | Refractory anaemia with excess blasts-2 (RAEB-2) |
|  |  |  | Refractory cytopenia with multilineage dysplasia (RCMD) |
| others | **others** | **others** | CD40 Ligand Deficiency |
|  |  |  | Chronic granulomatous disease (CGD) |
|  |  |  | Congenital Neutropenia (CN) |
|  |  |  | Ewings sarcoma |
|  |  |  | Haemoglobinopathy |
|  |  |  | Juvenile Chronic Arthritis (JIC) |
|  |  |  | Shwachman-Bodian-Diamond-Syndrome (SBDS) |
|  |  |  | severe combined immunodeficiency (SCID) |
|  |  |  | Wiskott-Aldrich Syndrome (WAS) |
| Unknown | **unknown** | **unknown** |  |
|  |  |  | unspecified carcinoma |
|  |  |  | unknown |

MF was assumed to be Myelofibrosis (but not mycosis fungoides); MPF was assumed to be PMF; MLN was assumed to be MPN; records like “AML from MDS” or “MDS transition to AML” was assumed to be “sAML”

Therapy response at Transplantation (stage of base disease)

S-Table 6 Classification of stage at Tx (response to previous therapies)

| Stage Level 1 classification | Stage Level 2 classification | State coded as ... |
| --- | --- | --- |
| **Complete remission (CR)** | Complete remission (CR) | CR |
|  |  | CR1 |
|  |  | CR2 |
|  |  | CR2+ |
|  |  | CR3 |
|  |  | CR? |
|  | cytologic CR (CRc) | CRc |
|  | Hematologic CR (CRh) | CRh |
| **Partial remission (PR)** | any remission (?R) | ?R |
|  | any molecular remission (?Rm) | ?Rm |
|  | Partial remission (PR) | PR |
|  |  | PR1 |
|  |  | PR2 |
|  |  | PR2+ |
|  |  | PR3 |
|  | Very Good Partial Response (VGPR) | VGPR |
|  | Mixed response (mixedR) | mixedR |
| **Progressive or resistant disease (PD/RD)** | No Response/Stable Disease (NR/SD) | NR |
|  |  | SD |
|  | Progressive Disease (PD) | PD |
|  | Increase in disease burden or new sites of disease (Progression) | Progression |
|  | Resistant disease (RD) | RD |
|  | Recurrence of disease after CP (relapse) | relapse |
|  |  | relapse1 |
|  |  | relapse2 |
|  |  | relapse3 |
| **belonging to CML/MPS/MPN** | Accelerated phase (AP) | AP |
|  |  | AP1 |
|  |  | AP? |
|  | Blast Phase/Crisis (BP) | BP |
|  | Chronic Phase (CP) | CP |
|  |  | CP1 |
|  |  | CP2 |
|  | high risk | high risk |
|  | intermediate risk | interm risk |
|  | low risk | low risk |
| **belonging to MDS** | unclassifiable MDS (MDS-U) | MDS |
|  | MDS with excess blasts (MDS-EB) | MDS-EB |
|  |  | MDS-EB1 |
|  |  | MDS-RAEB |
|  | MDS-RS with single lineage dysplasia (MDS-RS-SLD) | MDS-RA |
|  | Hypoplastic MDS (MDS-h) | MDS-h |
| **Untreated** | untreated | untreated |

# Cause of death

It was agreed to consider **any medical and social-medical causes of death** (including suicide) as **transplant-related** (if recorded and regardless any previous assignment of transplant related mortality). Survival times with unknown (not documented) causes of death, and non-medical/non-social-medical (e.g. found dead) will be considered as censored observation.

# Recoded conditioning-substances

Antibodies: Alemtuzumab, Anti-Lymphocyte-Globulin, Anti-Thymocyte-Globulin, Ibritumomab-Tiuxetan

Immunosuppressants: Cyclosporin, Methylprednisolone,

Cytostatics:

Alkylants: Busulphan, Carmustine, Cyclophosphamide, Melphalan, Thiotepa, Treosulfan,

Antimetabolite: Cytarabine, Fludarabine, Methotrexate

Topoisomerase inhibitor: Etoposide, Idarubicin

other cytostatic: Amsacrine, Mitoxantrone

Others: Glycoprotein, Mesna, Phenytoin

# Overall survival (OS) after onset of GvHD

To define a common GvHD-related outcome, we examined overall survival (OS) separately by GvHD stage diagnosed at the time of GvHD onset (see S-Figure 2). We examined the time from GvHD onset to death with a proportional hazard model among those 1,324 patients who were diagnosed with GvHD. The analysis was censored by relapse, adjusted by times from diagnosis to Tx, and Tx to GvHD, and conditionally estimated by study centre. The risk of dying is significantly higher with grade III or IV aGvHD than with grade I or II aGvHD (all p<0.0001, see S-Table 7).

S-Table 7 Survival after onset of GvHD: Hazard ratios by aGvHD grade

|  | proportional hazard model | | | Kaplan-Meier model | |
| --- | --- | --- | --- | --- | --- |
|  | HR | 95% CI | p-value | median  survival time | 95% CI |
| aGvHD grade 1 |  | reference |  | 5664 | 4547-unest. |
| aGvHD grade 2 vs 1 | 1.08 | 0.80-1.47 | 0.6071 | unest. | unest. |
| aGvHD grade 3 vs 1 | 2.63 | 1.90-3.65 | <.0001 | 526 | 222-1599 |
| aGvHD grade 4 vs 1 | 7.28 | 5.12-10.35 | <.0001 | 118 | 70-146 |
| cGvHD grade vs 1 | 1.20 | 0.86-1.68 | 0.2839 | 3634 | 3100-unest. |
| aGvHD grade 1/2 |  | reference |  | 5664 | 4547-unest. |
| sGvHD vs aGvHD grade 1/2 | 2.31 | 1.84-2.89 | <.0001 | 3100 | 1599-unest. |
| waiting-time [per month] | 0.997 | 0.995-1.000 | 0.0205 |  |  |
| GvHD time [per month] | 0.99 | 0.97-1.01 | 0.1489 |  |  |

95% CI 95% confidence interval; sGvHD aGvHD grade 3/4 or cGvHD
Proportional hazard model: HR hazard rate, adjusted for waiting-time: time from diagnosis to Tx and GvHD time: time from Tx to onset of GvHD; conditional to study centre; Kaplan-Meier model (unadjusted): median survival time; unest. cannot be estimated from the data

S-Figure 2: Survival after onset of GvHD: survival function by aGvHD


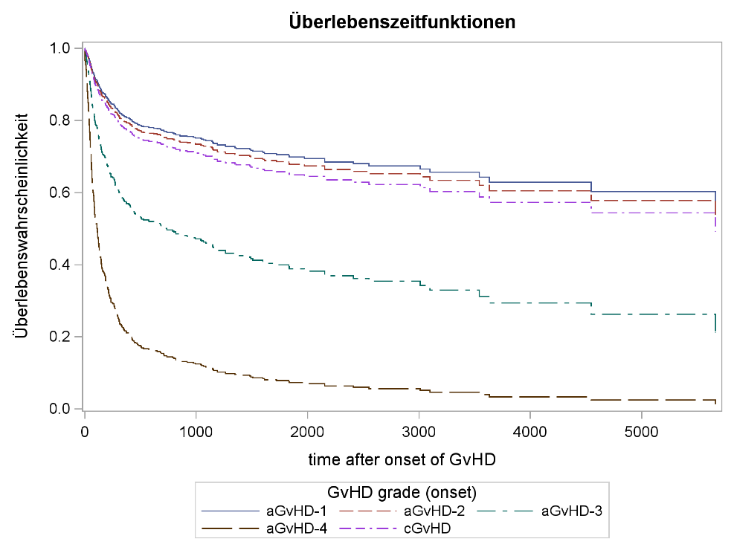


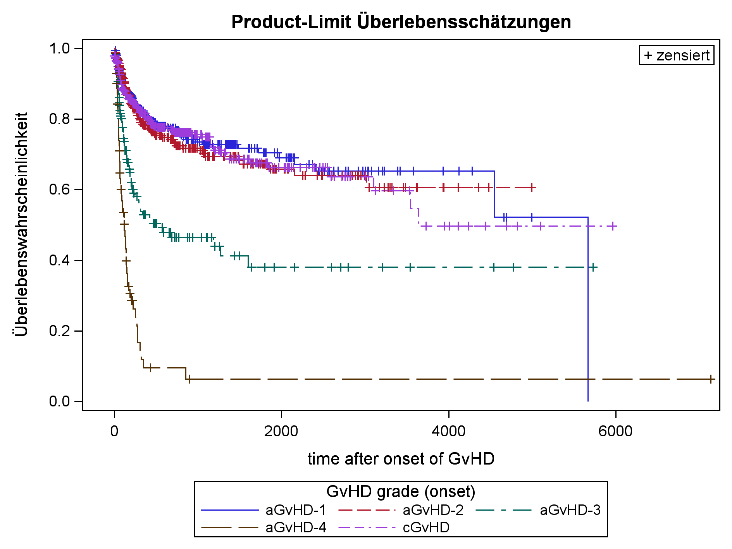


+ censord

left panel: proportional hazard model adjusted for waiting-time: time from diagnosis to Tx and GvHD time: time from Tx to onset of GvHD conditional to study centre; right panel: Kaplan-Meier Model (unadjusted)

# OS and EFS

S-Figure 3: Survival and cumulative hazard functions

| survival function | |  |
| --- | --- | --- |
| 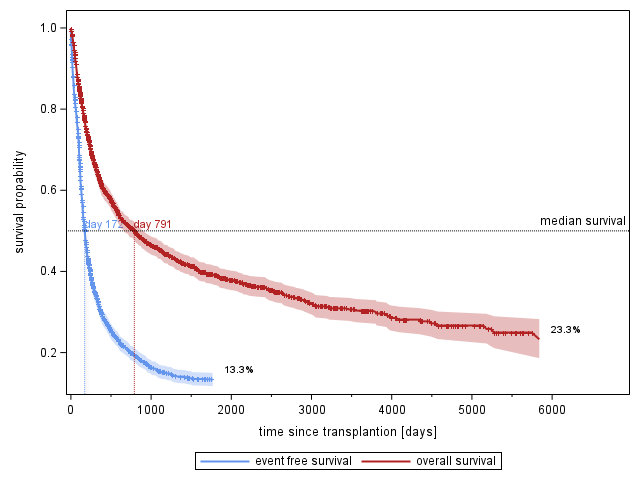 | | |
| cumulative hazard function: EFS | cumulative hazard function: OS |  |
| 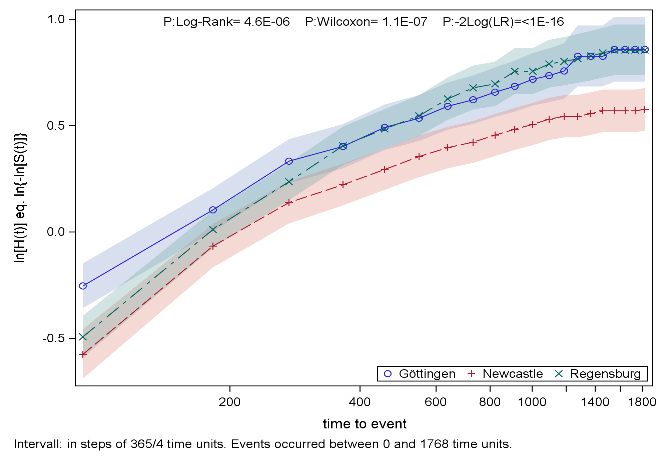 | 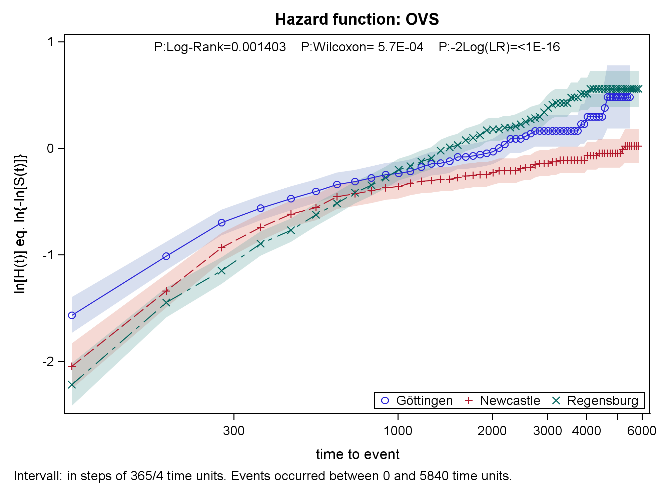 |  |

OS overall survival, EFS event-free survival, cumulative hazard function: all p-values of three different statistical tests (log-rank, Wilcoxon and -2log(LR)) show differences in cumulative hazard between study centre patient samples.

S-Table 8 Median survival times: OS and EFS

|  | | **time [day]** | **95% CI** | **yrs.** | **S(t)** | **95% CI** |
| --- | --- | --- | --- | --- | --- | --- |
| **EFS** | **25%** | 83 | 74-91 | 1 | 30% | 28%-32% |
|  | **50%** | 172 | 162-181 | 5 | 13% | 12%-15% |
|  | **75%** | 507 | 454-588 |  |  |  |
| **OS** | **25%** | 187 | 171-206 | 1 | 62% | 60%-64% |
|  | **50%** | 789 | 662-890 | 5 | 39% | 37% -1% |
|  | **75%** | -- | 4,349- | 10 | 30% | 27%-33% |

OS overall survival time, EFS event free survival; S(t) survival probability (years); 95% CI 95% confidence interval

# References

1. Amos CI, Dennis J, Wang Z, Byun J, Schumacher FR, Gayther SA, Casey G, Hunter DJ, Sellers TA, Gruber SB, et al. The OncoArray Consortium: A Network for Understanding the Genetic Architecture of Common Cancers. *Cancer Epidemiol Biomarkers Prev* (2017) 26:126–135. doi: 10.1158/1055-9965.EPI-16-0106
