# Supplementary material for: A genome-wide association study on hematopoietic stem cell transplantation reveals novel genomic loci associated with transplant outcomes
Source: Front Immunol. 2024 Feb 7;15:1280876. doi: 10.3389/fimmu.2024.1280876 (PMC10879589; doi:10.3389/fimmu.2024.1280876)
Supplement: Supplementary file 2 [file DataSheet_2.docx]

Supplement 2 (Modeling)

to: A genome-wide association study on hematopoietic stem cell transplantation reveals novel genomic loci associated with GvHD and Relapse
Albert Rosenberger et al.

# Unreliable parameter estimates

In this mass model fitting, unreliable parameter estimates can occur due to the numerous covariates in the model function and a small number of allele carriers (with a low MAF). We excluded results from further consideration if standard errors (s.e.) were large (s.e.>50), HR>1000 or HR<0.0001 or confidence interval were extremely wide (boundaries >10^5^ or <10^-5^).

# Pre-modelling / Screening Step

The “pre-models” were fitted for the whole sample (EFS, OS, relapse, NRGM and sGvHD) and subsamples of leukaemia, lymphoma, HLA-matched related donor, HLA-matched unrelated donor, Göttingen, Newcastle and Regensburg (EFS, OS and sGvHD). We generated QQ-plots to examine the distribution of p-values optically and estimated the genomic inflation factor λ, to robustly select apparent genome-wide significant SNPs (p≤10^−7^, gwSNPs) for more adequate modelling, as described above. Scans were excluded if λ>1.10, or when inadequacy of p-values was visually apparent. We also selected blocks of suggestive significant SNPs (10^−5^≤p≤ 10^−7^; suggSNPs) when in LD to each other (r²>0.8). We further selected all SNPs assigned to the same genes as gw/suggSNPs. We excluded HR estimates, as described above.

Only 38 of these 58 gwSNPs and 123 suggSNPs (21% of together 181 SNPs) are listed, due to at least one apparent association in the total sample, while 110 SNPs (60%) were exclusively selected, due to an apparent association in only one study centre (subgroup: Göttingen, Newcastle, Regensburg). Multiple apparent associations were the reason of selection for only 19 SNPs (21%; 6 gwSNPs and 13 suggSNPs). An apparent association to EFS was the reason for selection of 80 SNPs (44%), followed by OS of 51 SNPs (28%) and sGvHD of 48 SNPs (26%). Most of the selected SNPs (n=73), but together only 40%, are annotated to protein-coding genes, while 66 (36%) located in intergenic regions. Out of the 58 gwSNPs (33%), 19 had an MAF<1%.

## Score building for fast computation

S-Table 9 Estimates for scores: whole sample / AIC and fully adjusted models

| **model selection  / estimates for score building** | | **EFS** | | | | **OS** | | | |
| --- | --- | --- | --- | --- | --- | --- | --- | --- | --- |
|  |  | **AIC-best** | | **full-adjusted** | | **AIC-best** | | **full-adjusted** | |
|  |  | **β** | **p-value** | **β** | **p-value** | **β** | **p-value** | **β** | **p-value** |
| **P/D relationship** | **FdMp** | 0.2162 | 0.0004 | 0.2269 | 0.0006 | 0.2573 | 0.0006 | 0.2652 | 0.0012 |
| **GvHD Prophylaxes** | **yes** | -- | -- | -.1395 | 0.5196 | -.2375 | 0.3610 | -.2516 | 0.3365 |
| **ln(waiting time)** | **Unit** | -- | -- | -.0022 | 0.9345 | -- | -- | -.0111 | 0.7427 |
| **Patient’s age** | **Unit** | 0.0043 | 0.0535 | 0.0043 | 0.0611 | 0.0134 | <.0001 | 0.0135 | <.0001 |
| **Patient’s gender** | **female** | -- | -- | 0.0233 | 0.6612 | -- | -- | 0.0146 | 0.8280 |
| **Previous auto Tx** | **unknown** | -.5021 | <.0001 | -.4991 | <.0001 | -- | -- | 0.1081 | 0.3750 |
|  | **yes** | 0.1767 | 0.0209 | 0.1880 | 0.0295 | 0.2068 | 0.0386 | 0.2292 | 0.0326 |
| **RIC** | **yes** | -- | -- | -.0159 | 0.8230 | -- | -- | -.0480 | 0.5753 |
| **TBI** | **yes** | 0.2232 | 0.0022 | 0.2205 | 0.0052 | 0.1697 | 0.0302 | 0.1537 | 0.1032 |
| **T-cell depletion** | **yes** | -.2552 | 0.0007 | -.2515 | 0.0009 | -.0798 | 0.3217 | -.1135 | 0.2307 |
| **underlying condition** | **lymphoma** | -- | -- | -.0168 | 0.8059 | -.1470 | 0.0503 | -.1705 | 0.0457 |
| **stage at Tx** | **CML/MPS/MPN** | 0.2542 | 0.0194 | 0.2557 | 0.0206 | 0.1973 | 0.1189 | 0.2222 | 0.0961 |
|  | **MDS** | -- | -- | 0.0360 | 0.7785 | -- | -- | -.1080 | 0.5388 |
|  | **PD/PR** | 0.4678 | <.0001 | 0.4756 | <.0001 | 0.6454 | <.0001 | 0.6590 | <.0001 |
|  | **PR** | 0.1405 | 0.0330 | 0.1527 | 0.0285 | 0.2163 | 0.0078 | 0.2329 | 0.0081 |
|  | **CR** | 0.4143 | 0.0002 | 0.4169 | 0.0003 | 0.5373 | <.0001 | 0.5494 | <.0001 |
|  | **others** | -.0630 | 0.5212 | -.0570 | 0.5679 | -- | -- | 0.1092 | 0.3902 |
| **Conditioning with** |  |  |  |  |  |  |  |  |  |
| TI | **yes** | 0.4688 | 0.0114 | 0.4608 | 0.0134 | -- | -- | 0.0878 | 0.7126 |
| alkylate | **yes** | 0.0694 | 0.4660 | 0.0715 | 0.4574 | -- | -- | -.0005 | 0.9963 |
| antibody | **yes** | 0.2414 | 0.0034 | 0.2390 | 0.0041 | -- | -- | 0.0831 | 0.4076 |
| antimetabolite | **yes** | 0.1717 | 0.0276 | 0.1806 | 0.0338 | -- | -- | -.0200 | 0.8427 |
| other | **yes** | 0.1344 | 0.2367 | 0.1313 | 0.2657 | -- | -- | 0.0444 | 0.7678 |
| **graft** | **BM** | -.3115 | 0.0005 | -.3142 | 0.0004 | -- | -- | -.0685 | 0.4947 |
|  | **CB** | -- | -- | 0.0481 | 0.8970 | 0.8850 | 0.0164 | 0.8459 | 0.0321 |
|  | **PBSC** | 0.9915 | 0.0281 | 0.9927 | 0.0282 | 1.2106 | 0.0155 | 1.2035 | 0.0165 |
| **relationship** | **MRD (inc. SIBs)** | -.1363 | 0.0301 | -.1350 | 0.0337 | -.2119 | 0.0071 | -.2097 | 0.0098 |
|  | **miscellaneous** | -.1214 | 0.4654 | -.1451 | 0.4269 | 0.1896 | 0.3616 | 0.1555 | 0.4630 |

β estimated coefficient in a COX-model, EFS event free survival, OS overall survival, RIC reduced intensive conditioning, TBI previous total body irradiation, TI Topoisomerase inhibitor, MRD HLA-matched related donor, BM bone marrow, CB cord blood, PBSC peripheral blood stem cells, CML belonging to CML/MPS/MPN, MDS belonging to MDS, PD progressive or resistant disease, PR partial remission, CR complete remission, FdMp female donor to male recipient

We performed 52 fast genome-wide scans for SNP associated with EFS, OS or time to a key event, in the total cohort and in 7 subcohorts, crude and adjusted for scores composed on covariates. Genomic inflation (λ>1.10) or visually apparent inadequate p-values were achieved in six of these scans, which were excluded for SNP selection.

For six out of 52 pre-scans a genomic inflation of λ>1.10 or apparently inadequate p-values were achieved (see S-Table 10). Based on the remaining 46 pre-scans, we selected for more adequate modelling 58 gwSNPs (p≤10^−7^) and 123 suggSNPs (of multi-marker LD blocks), among others (see S-Table 11). Only six SNPs were apparently associated in more than one subsample, thus showing some consistency. These were rs2732922 (2p24.2, p=8.7x10−7, intronic of *KCNS3*) and rs2517582 (6p21.33, p=4.0x10^−7^, intronic between XXbac-BPG27H4.8 and LINC02570). Both markers are common variants (MAF≥5%) and appeared associated to OS e.g. in the total sample (not only in a subsample). The four other multi-associated gwSNPs (rs34863545, rs116369879, rs6949036, rs35491243) have rare variants (MAF<5%) and are located in intergenic regions.

Worth mentioning is the only LD-pair of gwSNPs associated in the total sample. Both gwSNPs (rs1151832, rs4767886) are located intronic of gene *PXN* (12q24.23), and are in strong LD to each other (D'=1, R²=0.9498). They are apparently associated to NRGM (p=4.5x10^-7^ and p=1.5x10^-7^).

We further observed three LD-blocks of apparently associated markers. One is a block between 6q22.1 and 6q23 (comprising 24 SNPs, covering the HLA region), another LD-block located at 4q32.2, comprised of four common, but intergenic markers. Further, there was a block of five common markers (D’=1 but low R²), located at 8q24.21 around the gene *PVT1*, suggestive of association with EFS or sGvHD, in the either the total sample or in leukaemia or lymphoma patients.

S-Table 10 Genomic inflation factor λ

| subsample | category | size of subsample |  | EFS crude | EFS adjusted | OS crude | OS adjusted |
| --- | --- | --- | --- | --- | --- | --- | --- |
| **Total sample** |  | 1392 | any | λ=1,01 | λ=1,08 | λ=1,00 | λ=1,04 |
|  |  |  | NRGM | λ=1,01 | λ=1,03 | -- | -- |
|  |  |  | relapse | λ=1,02 | λ=1,02 | -- | -- |
|  |  |  | sGvHD | λ=1,08^$^ | λ=1,03 | -- | -- |
| **Underlying condition (disease)** | leukaemia | 882 |  | λ=1,01 | λ=1,08 | λ=1,00 | λ=1,04 |
|  |  |  | sGvHD | λ=1,08^$^ | λ=1,03 | -- | -- |
|  | lymphoma | 473 |  | λ=1,02^$^ | λ=1,08 | λ=1,01 | λ=1,06 |
|  |  |  | sGvHD | λ=1,02 | λ=1,03 | -- | -- |
| **Patient/Donor relationship** | HLA-matched related donor | 204 |  | λ=1,01 | λ=1,08 | λ=1,00 | λ=1,04 |
|  |  |  | sGvHD | λ=1,08 | λ=1,03 | -- | -- |
|  | HLA-matched unrelated donor | 950 |  | λ=1,01 | λ=1,08 | λ=1,00 | λ=1,04 |
|  |  |  | sGvHD | λ=1,01 | λ=1,03 | -- | -- |
| **Study centre** | Göttingen | 548 | any | λ=1,01 | λ=1,06 | λ=1,00 | λ=1,02 |
|  |  |  | sGvHD | λ=1,01 | λ=1,01 | -- | -- |
|  | Newcastle | 258 |  | λ=0,99 | λ=1,17 | λ=1,02 | λ=1,07 |
|  |  |  | sGvHD | λ=1,03 | λ=1,10 | -- | -- |
|  | Regensburg | 586 |  | λ=1,00 | λ=1,12 | λ=1,03 | λ=1,10 |
|  |  |  | sGvHD | λ=1,10 | λ=1,10^$^ | -- | -- |

^$^ Inadequacy of p-values visually apparent

## SNP-selection by Screening Step

S-Table 11 Characteristic of SNPs selected in the screening step

| **gene type / SNP function** | **total SNPs** | | **reason of selection** | | | | | | | | | | | |
| --- | --- | --- | --- | --- | --- | --- | --- | --- | --- | --- | --- | --- | --- | --- |
|  |  |  | **cluster SNPs** | | | | | | **gw-sig SNPs** | | | | | |
|  |  |  | **all** | | **coding** | **intergenic** | **intronic** | **up/downstream** | **all** | | **coding** | **intergenic** | **intronic** | **up/downstream** |
| **total** | 181 | 100% | 123 | 100% | 6 | 41 | 65 | 11 | 58 | 100% | 1 | 25 | 29 | 3 |
| **antisense** | 9 | 5% | 5 | 4% | 2 | -- | 2 | 1 | 4 | 7% | -- | -- | 3 | 1 |
| **intergenic** | 66 | 36% | 41 | 33% | -- | 41 | -- | -- | 25 | 43% | -- | 25 | -- | -- |
| **lincRNA** | 16 | 9% | 11 | 9% | -- | -- | 11 | -- | 5 | 9% | -- | -- | 5 | -- |
| **processed transcript** | 6 | 3% | 5 | 4% | -- | -- | 5 | -- | 1 | 2% | -- | -- | 1 | -- |
| **protein coding** | 73 | 40% | 50 | 41% | 1 | -- | 39 | 10 | 23 | 40% | 1 | -- | 20 | 2 |
| **pseudogene** | 9 | 5% | 9 | 7% | 3 | -- | 6 | -- | -- | -- | -- | -- | -- | -- |
| **sense intronic** | 2 | 1% | 2 | 2% | -- | -- | 2 | -- | -- | -- | -- | -- | -- | -- |
| **# close SNPs** |  |  |  |  |  |  |  |  |  |  |  |  |  |  |
| 0 | 54 | 30% | -- | -- | -- | -- | -- | -- | 54 | 93% | 1 | 25 | 25 | 3 |
| 1 | 111 | 61% | 107 | 87% | 3 | 38 | 55 | 11 | 4 | 7% | -- | -- | 4 | -- |
| 2 | 11 | 6% | 11 | 9% | 1 | 1 | 9 | -- | -- | -- | -- | -- | -- | -- |
| 4 | 5 | 3% | 5 | 4% | 2 | 2 | 1 | -- | -- | -- | -- | -- | -- | -- |
| **MAF (European CEU)** |  |  |  |  |  |  |  |  |  |  |  |  |  |  |
| -- | 1 | <1% | 1 | <1% | -- | -- | 1 | -- | -- | -- | -- | -- | -- | -- |
| <1% | 22 | 12% | 3 | 2% | -- | 1 | 2 | -- | 19 | 33% | 1 | 9 | 8 | 1 |
| 1%-5% | 24 | 13% | 14 | 11% | -- | -- | 10 | 4 | 10 | 17% | -- | 6 | 3 | 1 |
| 5%-10% | 23 | 13% | 14 | 11% | 2 | 2 | 8 | 2 | 9 | 16% | -- | 2 | 7 | -- |
| 10%-50% | 111 | 61% | 91 | 74% | 4 | 38 | 44 | 5 | 20 | 34% | -- | 8 | 11 | 1 |

S-Table 12 SNP-selection (pre-modelling): genome-wide sig. SNPs

| **SNP** | **location** | **MAF** | **annotated gene(s)** | **gene type** | **predicted SNP function** | **min. p-value** | **# close SNPs** | **# subgroups** | **subgroups  in which the SNP was significant** | **key event** |
| --- | --- | --- | --- | --- | --- | --- | --- | --- | --- | --- |
| rs12029475 | 1p33 | 3% | ELAVL4 | protein coding | intronic | 8.4E-07 | 0 | 1 | LYMPHOMA | EFS |
| rs2174689 | 1p36.12 | 6% | EIF4G3 | protein coding | intronic | 3.2E-08 | 0 | 1 | NEWCASTLE | OS |
| rs34599082 | 1q23.2 | 0% | CTA-134P22.2 | protein coding | intronic | 3.9E-07 | 0 | 1 | NEWCASTLE | OS |
| rs140249458 | 1q23.3 | 1% | ATF6 | protein coding | intronic | 5.5E-07 | 0 | 1 | NEWCASTLE | sGvHD |
| rs77398593 | 1q41 | 0% | (RP11-400N13.3,AC092765.1 | intergenic | intergenic | 2.6E-07 | 0 | 1 | LYMPHOMA | EFS |
| rs2732922 | 2p24.2 | 15% | KCNS3 | protein coding | intronic | 8.7E-07 | 0 | 3 | ALL RELATED UNRELATED | OS |
| rs35491243 | 2q35 | 0% | (AC010887.1,RP11-574O16.1 | intergenic | intergenic | 2.6E-07 | 0 | 4 | ALL RELATED LEUKEMIA UNRELATED | EFS |
| rs1678100 | 3p14.1 | 10% | SLC25A26 | protein coding | intronic | 5.7E-07 | 0 | 1 | REGENSBURG | EFS |
| rs75976589 | 3p25.1 | 0% | (AC090952.5,RHBDF1P1) | intergenic | intergenic | 2.3E-07 | 0 | 1 | GOTTINGEN | sGvHD |
| rs13066393 | 3p26.3 | 23% | AC090044.1 | lincRNA | intronic | 4.0E-07 | 0 | 1 | GOTTINGEN | EFS |
| rs116369879 | 3q26.1 | 0% | (RP11-71H9.2,MIR720) | intergenic | intergenic | 5.3E-07 | 0 | 4 | ALL RELATED LEUKEMIA UNRELATED | EFS |
| rs76271330 | 4p12 | 1% | TXK | protein coding | intronic | 4.8E-07 | 0 | 1 | GOTTINGEN | OS |
| rs149502180 | 4q21.1 | 0% | RP11-359D14.3 | antisense | intronic | 2.5E-07 | 0 | 1 | GOTTINGEN | sGvHD |
| rs1354681 | 4q25 | 14% | ANK2 | antisense | intronic | 2.1E-07 | 0 | 1 | NEWCASTLE | EFS |
| rs12521066 | 5q14.1 | 25% | (AP3B1,RP11-107N7.1) | intergenic | intergenic | 5.6E-07 | 0 | 1 | NEWCASTLE | OS |
| rs145328468 | 5q33.3 | 0% | EBF1 | protein coding | intronic | 6.5E-07 | 0 | 1 | NEWCASTLE | OS |
| rs7714271 | 5q35.2 | 6% | CTB-43E15.2 | lincRNA | intronic | 2.7E-07 | 0 | 1 | NEWCASTLE | sGvHD |
| rs2517582 | 6p21.33 | 29% | XXbac-BPG27H4.8 | lincRNA | intronic | 4.0E-07 | 1 | 5 | ALL UNRELATEDTED LEUKEMIA RELATED UNRELATED | OS |
| rs56154906 | 6p21.33 | 3% | (XXbac-BPG27H4.8,LINC0024 | intergenic | intergenic | 7.8E-07 | 0 | 1 | NEWCASTLE | EFS |
| rs138553412 | 7p21.1 | 1% | ABCB5 | protein coding | up/downstream | 1.9E-08 | 0 | 1 | GOTTINGEN | sGvHD |
| rs1525745 | 7p21.1 | 1% | (RP11-455J15.1,AGR2) | intergenic | intergenic | 1.9E-07 | 0 | 1 | GOTTINGEN | sGvHD |
| rs80026286 | 7p21.3 | 1% | (AC079756.1,RP4-668E10.2) | intergenic | intergenic | 6.4E-08 | 0 | 1 | NEWCASTLE | OS |
| rs6949036 | 7q36.2 | 6% | (AC073236.3,AC079809.2) | intergenic | intergenic | 4.3E-07 | 0 | 4 | ALL RELATED LEUKEMIA UNRELATED | sGvHD |
| rs74552627 | 8p21.2 | 1% | RP11-624C23.1 | antisense | intronic | 3.2E-08 | 0 | 1 | LYMPHOMA | sGvHD |
| rs34863545 | 8p22 | 1% | (NATP,NAT1) | intergenic | intergenic | 6.0E-07 | 0 | 4 | ALL RELATED LEUKEMIA UNRELATED | sGvHD |
| rs10102694 | 8q12.1 | 7% | (RNA5SP267,SNORA51) | intergenic | intergenic | 8.4E-07 | 0 | 1 | REGENSBURG | EFS |
| rs55949743 | 8q12.1 | 12% | RP11-246K15.1 | lincRNA | intronic | 9.9E-07 | 1 | 1 | NEWCASTLE | OS |
| rs7830834 | 8q12.1 | 28% | (RNA5SP267,SNORA51) | intergenic | intergenic | 2.6E-08 | 0 | 1 | REGENSBURG | EFS |
| rs28907868 | 8q21.3 | 2% | RP11-586K2.1 | antisense | up/downstream | 7.9E-07 | 0 | 1 | GOTTINGEN | OS |
| rs62531502 | 8q24.3 | 4% | (ZNF517,RPL8) | intergenic | intergenic | 3.9E-07 | 0 | 1 | LYMPHOMA | sGvHD |
| rs16932807 | 9p13.3 | 3% | (YBX1P10,OR2S2) | intergenic | intergenic | 4.8E-07 | 0 | 1 | REGENSBURG | EFS |
| rs147949683 | 9q34.3 | 0% | (SOCS5P2,RP11-555H7.2) | intergenic | intergenic | 3.9E-07 | 0 | 1 | NEWCASTLE | sGvHD |
| rs353206 | 10p14 | 39% | (LINC00707,RP11-554I8.2) | intergenic | intergenic | 8.1E-07 | 0 | 1 | REGENSBURG | EFS |
| rs7919925 | 10p14 | 0% | SFMBT2 | protein coding | intronic | 4.3E-08 | 0 | 1 | NEWCASTLE | sGvHD |
| rs74707950 | 10q21.3 | 3% | EGR2 | protein coding | intronic | 3.2E-07 | 0 | 1 | NEWCASTLE | OS |
| rs34588967 | 10q22.3 | 1% | POLR3A | protein coding | coding | 1.3E-07 | 0 | 1 | ALL | NRGM |
| rs7928641 | 11q14.1 | 9% | RAB30 | protein coding | intronic | 6.1E-07 | 0 | 1 | LYMPHOMA | EFS |
| rs75029885 | 11q22.2 | 1% | BIRC2 | protein coding | intronic | 5.7E-08 | 0 | 1 | GOTTINGEN | EFS |
| rs1151832 | 12q24.23 | 39% | PXN | protein coding | intronic | 4.5E-07 | 1 | 1 | ALL | NRGM |
| rs4767886 | 12q24.23 | 22% | PXN | protein coding | intronic | 1.5E-07 | 1 | 1 | ALL | NRGM |
| rs76623214 | 13q13.1 | 0% | PDS5B | protein coding | intronic | 1.8E-07 | 0 | 1 | LYMPHOMA | sGvHD |
| rs9516483 | 13q32.1 | 0% | (LINC00557,RPL21P112) | intergenic | intergenic | 9.4E-07 | 0 | 1 | REGENSBURG | EFS |
| rs16959817 | 15q21.1 | 5% | SEMA6D | protein coding | intronic | 3.1E-07 | 0 | 1 | NEWCASTLE | EFS |
| rs1990230 | 15q26.1 | 5% | SLCO3A1 | protein coding | intronic | 6.6E-07 | 0 | 1 | REGENSBURG | EFS |
| rs116128588 | 16q24.1 | 3% | (FENDRR,RP11-158I3.1) | intergenic | intergenic | 6.2E-09 | 0 | 1 | LYMPHOMA | EFS |
| rs62053951 | 17q21.31 | 9% | CRHR1 | processed transcript | intronic | 3.4E-07 | 0 | 1 | ALL | RELAPSE |
| rs3785912 | 17q21.33 | 29% | ABCC3 | protein coding | intronic | 8.8E-08 | 0 | 1 | GOTTINGEN | EFS |
| rs8085108 | 18q12.2 | 18% | (RP11-322E11.5,RP11-322E1 | intergenic | intergenic | 5.8E-07 | 0 | 1 | LYMPHOMA | sGvHD |
| rs2288408 | 19p13.11 | 15% | MVB12A | protein coding | up/downstream | 9.8E-07 | 0 | 1 | GOTTINGEN | OS |
| rs16964067 | 19q12 | 31% | ZNF536 | protein coding | intronic | 6.1E-07 | 0 | 1 | GOTTINGEN | EFS |
| rs9630865 | 19q12 | 43% | ZNF536 | protein coding | intronic | 4.4E-07 | 0 | 1 | GOTTINGEN | EFS |
| rs2827415 | 21q21.1 | 9% | AP000705.7 | lincRNA | intronic | 1.2E-07 | 0 | 1 | NEWCASTLE | sGvHD |
| rs9980706 | 21q21.2 | 18% | (Y_RNA,TUBAP) | intergenic | intergenic | 9.5E-07 | 0 | 1 | NEWCASTLE | sGvHD |
| rs743802 | 22q13.33 | 32% | (RPL35P8,WI2-81516E3.1) | intergenic | intergenic | 9.4E-07 | 0 | 1 | GOTTINGEN | EFS |
| rs139378727 | Xp21.3 | 0% | (RP11-268G12.3,RNU1-142P) | intergenic | intergenic | 2.6E-09 | 0 | 1 | REGENSBURG | EFS |
| rs5978996 | Xp22.31 | 40% | (FAM9B,RP11-66N5.2) | intergenic | intergenic | 2.9E-09 | 0 | 1 | LYMPHOMA | EFS |
| rs3811374 | Xq13.1 | 24% | (EDA,RP13-57D9.3) | intergenic | intergenic | 5.2E-07 | 0 | 1 | NEWCASTLE | OS |
| rs144389131 | Xq12 | 0% | (RNU6-394P,EDA2R) | intergenic | intergenic | 2.4E-07 | 0 | 1 | NEWCASTLE | sGvHD |

S-Table 13 SNP-selection (preScan): clustered, suggestive sig. SNPs

| **marker (SNP)** | **cytogenetic location** | **MAF** | **annotated gene(s)** | **gene type** | **predicted SNP function** | **Min. p-value** | **# close SNPs** | **# subgroups** | **subgroups  in which the SNP was significant** | **key event** |
| --- | --- | --- | --- | --- | --- | --- | --- | --- | --- | --- |
| rs854273 | 1p31.2 | 21% | (RP11-424D14.1,RP4-694A7.4) | intergenic | intergenic | 6.5E-06 | 1 | 1 | NEWCASTLE | EFS |
| rs6692084 | 1p31.2 | 29% | (RP11-424D14.1,RP4-694A7.4) | intergenic | intergenic | 2.7E-06 | 1 | 1 | NEWCASTLE | EFS |
| rs380337 | 1p36.11 | 37% | RP4-799D16.1 | lincRNA | intronic | 9.9E-05 | 1 | 1 | GOTTINGEN | OS |
| rs6679217 | 1p36.11 | 29% | RP11-223J15.2 | pseudogene | intronic | 5.3E-05 | 1 | 1 | NEWCASTLE | EFS |
| rs7549155 | 1p36.11 | 27% | (RP11-223J15.2,MDS2) | intergenic | intergenic | 4.1E-05 | 1 | 1 | NEWCASTLE | EFS |
| rs443523 | 1p36.11 | 28% | RP4-799D16.1 | lincRNA | intronic | 6.6E-06 | 1 | 1 | GOTTINGEN | OS |
| rs35219978 | 1q32.1 | 46% | (MDM4,PIK3C2B) | intergenic | intergenic | 7.8E-05 | 1 | 1 | GOTTINGEN | OS |
| rs4951389 | 1q32.1 | 46% | (MDM4,PIK3C2B) | intergenic | intergenic | 7.8E-05 | 1 | 1 | GOTTINGEN | OS |
| rs1341452 | 1q43 | 47% | (RP11-527D7.1,RGS7) | intergenic | intergenic | 9.4E-05 | 1 | 1 | REGENSBURG | EFS |
| rs1341448 | 1q43 | 29% | (RP11-527D7.1,RGS7) | intergenic | intergenic | 7.2E-06 | 1 | 1 | REGENSBURG | EFS |
| rs59113870 | 20p12.1 | 8% | MACROD2 | sense intronic | intronic | 4.3E-06 | 1 | 1 | NEWCASTLE | sGvHD |
| rs8122992 | 20p12.1 | 7% | MACROD2 | sense intronic | intronic | 4.3E-06 | 1 | 1 | NEWCASTLE | sGvHD |
| rs142333882 | 2p16.2 | 1% | AC010967.2 | lincRNA | intronic | 6.1E-05 | 1 | 1 | NEWCASTLE | EFS |
| rs77138737 | 2p16.2 | 4% | AC010967.2 | lincRNA | intronic | 6.1E-05 | 1 | 1 | NEWCASTLE | EFS |
| rs2278719 | 2p21 | 7% | AC016735.1 | lincRNA | intronic | 5.8E-05 | 1 | 1 | NEWCASTLE | OS |
| rs3889781 | 2p21 | 38% | (AC016735.2,AC016735.1) | intergenic | intergenic | 5.1E-05 | 1 | 1 | NEWCASTLE | EFS |
| rs56350069 | 2p24.2 | 36% | (AC092594.1,AC106053.1) | intergenic | intergenic | 9.5E-05 | 1 | 1 | NEWCASTLE | sGvHD |
| rs6531120 | 2p24.2 | 38% | (AC092594.1,AC106053.1) | intergenic | intergenic | 7.8E-05 | 1 | 1 | NEWCASTLE | sGvHD |
| rs9286350 | 2p25.3 | 27% | SNTG2 | protein coding | intronic | 8.2E-05 | 1 | 1 | REGENSBURG | EFS |
| rs9712145 | 2p25.3 | 27% | SNTG2 | protein coding | intronic | 2.6E-05 | 1 | 1 | REGENSBURG | EFS |
| rs2055593 | 2q11.2 | 34% | CNGA3 | protein coding | intronic | 6.1E-05 | 1 | 1 | REGENSBURG | EFS |
| rs13424165 | 2q11.2 | 6% | CNGA3 | protein coding | intronic | 2.6E-05 | 1 | 1 | REGENSBURG | EFS |
| rs16832006 | 2q21.3 | 16% | (LCT,UBXN4) | intergenic | intergenic | 9.4E-05 | 1 | 1 | GOTTINGEN | sGvHD |
| rs4954633 | 2q21.3 | 24% | LCT | protein coding | intronic | 2.4E-05 | 1 | 1 | GOTTINGEN | EFS |
| rs7620921 | 3q26.1 | 17% | RP11-85M11.2 | lincRNA | intronic | 6.6E-05 | 1 | 5 | ALL RELATED UNRELATED LEUKEMIA NEWCASTLE | OS |
| rs10936456 | 3q26.1 | 13% | RP11-85M11.2 | lincRNA | intronic | 4.1E-05 | 1 | 1 | NEWCASTLE | OS |
| rs1553876 | 4p15.2 | 32% | ZCCHC4 | protein coding | intronic | 8.1E-05 | 1 | 1 | NEWCASTLE | sGvHD |
| rs10939051 | 4p15.2 | 42% | ZCCHC4 | protein coding | intronic | 3.5E-05 | 1 | 1 | NEWCASTLE | sGvHD |
| rs6536880 | 4q32.3 | 34% | (RP11-366M4.8,NACA3P) | intergenic | intergenic | 7.5E-05 | 4 | 3 | ALL LEUKEMIA UNRELATED | EFS |
| rs4443238 | 4q32.3 | 32% | (NACA3P,RP11-366M4.6) | intergenic | intergenic | 3.4E-05 | 2 | 3 | ALL LEUKEMIA UNRELATED | EFS |
| rs4541460 | 4q32.3 | 34% | RP11-366M4.8 | pseudogene | coding | 3.4E-05 | 2 | 3 | ALL LEUKEMIA UNRELATED | EFS |
| rs4370095 | 4q32.3 | 36% | RP11-366M4.8 | pseudogene | coding | 3.3E-05 | 4 | 3 | ALL LEUKEMIA UNRELATED | EFS |
| rs4425332 | 4q32.3 | 36% | RP11-366M4.8 | pseudogene | coding | 3.3E-05 | 4 | 3 | ALL LEUKEMIA UNRELATED | EFS |
| rs6536881 | 4q32.3 | 34% | (RP11-366M4.8,NACA3P) | intergenic | intergenic | 2.5E-05 | 4 | 3 | ALL LEUKEMIA UNRELATED | EFS |
| rs4621476 | 4q34.3 | 45% | (RP11-433O3.1,RP11-665C14 | intergenic | intergenic | 4.8E-05 | 1 | 1 | NEWCASTLE | sGvHD |
| rs4861947 | 4q34.3 | 26% | (RP11-433O3.1,RP11-665C14 | intergenic | intergenic | 8.8E-06 | 1 | 1 | NEWCASTLE | sGvHD |
| rs1877840 | 5p15.33 | 38% | AHRR | protein coding | intronic | 3.6E-05 | 1 | 1 | LYMPHOMA | OS |
| rs908114 | 5p15.33 | 44% | AHRR | protein coding | intronic | 6.9E-06 | 1 | 1 | LYMPHOMA | OS |
| rs6453484 | 5q14.1 | 29% | CMYA5 | antisense | coding | 4.8E-06 | 1 | 1 | LYMPHOMA | sGvHD |
| rs9283795 | 5q14.1 | 10% | CMYA5 | antisense | coding | 3.7E-06 | 1 | 1 | LYMPHOMA | sGvHD |
| rs111549785 | 6p12.1 | 1% | (BMP5,RP11-228O6.2) | intergenic | intergenic | 6.8E-05 | 1 | 1 | GOTTINGEN | OS |
| rs80153042 | 6p12.1 | 1% | BMP5 | protein coding | up/downstream | 3.0E-06 | 1 | 1 | GOTTINGEN | OS |
| rs117340637 | 6q22.1 | 1% | ROS1 | protein coding | intronic | 6.7E-05 | 1 | 1 | LYMPHOMA | OS |
| rs200257785 | 6p21.32 | *24% | HLA-DRB9 | pseudogene | intronic | 7.6E-05 | 2 | 3 | ALL UNRELATED LEUKEMIA | OS |
| rs11364248 | 6p21.33 | 3% | XXbac-BPG27H4.8 | lincRNA | intronic | 4.1E-06 | 2 | 1 | NEWCASTLE | EFS |
| rs2844664 | 6p21.33 | 23% | (RN7SKP186,XXbac-BPG27H4.8) | intergenic | intergenic | 2.1E-06 | 1 | 5 | ALL UNRELATEDTED LEUKEMIA RELATED UNRELATED | OS |
| rs3131785 | 6p21.33 | 32% | (DPCR1,Y_RNA) | intergenic | intergenic | 4.0E-06 | 1 | 7 | ALL UNRELATEDTED LEUKEMIA RELATED UNRELATED GOTTINGEN LYMPHOMA | OS |
| rs3131784 | 6p21.33 | 32% | (DPCR1,Y_RNA) | intergenic | intergenic | 1.7E-06 | 1 | 7 | ALL UNRELATEDTED LEUKEMIA RELATED UNRELATED GOTTINGEN LYMPHOMA | OS |
| rs3219190 | 6p21.33 | 4% | MCCD1 | protein coding | up/downstream | 1.0E-05 | 1 | 1 | NEWCASTLE | sGvHD |
| rs3853601 | 6p21.33 | 10% | ATP6V1G2-DDX39B | protein coding | up/downstream | 1.6E-05 | 1 | 1 | NEWCASTLE | sGvHD |
| rs147405535 | 6p21.33 | 6% | SAPCD1-AS1 | antisense | up/downstream | 4.3E-05 | 1 | 1 | LYMPHOMA | OS |
| rs707936 | 6p21.33 | 12% | VWA7 | protein coding | up/downstream | 3.2E-05 | 1 | 4 | ALL UNRELATED LEUKEMIA LYMPHOMA | OS |
| rs9268918 | 6p21.32 | 29% | HLA-DRB9 | pseudogene | intronic | 7.6E-05 | 2 | 3 | ALL UNRELATED LEUKEMIA | OS |
| rs9268977 | 6p21.32 | 29% | HLA-DRB9 | pseudogene | intronic | 4.7E-05 | 4 | 3 | ALL UNRELATED LEUKEMIA | OS |
| rs9269043 | 6p21.32 | 29% | HLA-DRB9 | pseudogene | intronic | 9.4E-05 | 2 | 3 | ALL UNRELATED LEUKEMIA | OS |
| rs7452863 | 6p21.32 | 31% | HLA-DRB9 | pseudogene | intronic | 7.6E-05 | 2 | 3 | ALL UNRELATED LEUKEMIA | OS |
| rs140912153 | 6q22.1 | 1% | ROS1 | protein coding | intronic | 1.4E-05 | 2 | 1 | LYMPHOMA | OS |
| rs150750988 | 6q22.1 | 0% | ROS1 | protein coding | intronic | 1.5E-05 | 1 | 1 | LYMPHOMA | EFS |
| rs713215 | 6q22.31 | 20% | NKAIN2 | protein coding | intronic | 3.4E-06 | 1 | 5 | NEWCASTLE ALL LEUKEMIA RELATED UNRELATED | sGvHD |
| rs7771967 | 6q22.31 | 15% | NKAIN2 | protein coding | intronic | 6.0E-06 | 1 | 5 | NEWCASTLE ALL LEUKEMIA RELATED UNRELATED | sGvHD |
| rs12525126 | 6p23 | 18% | (MCUR1,RANBP9) | intergenic | intergenic | 2.3E-05 | 1 | 1 | LYMPHOMA | EFS |
| rs12665864 | 6p23 | 27% | (MCUR1,RANBP9) | intergenic | intergenic | 4.6E-05 | 1 | 1 | LYMPHOMA | EFS |
| rs12529521 | 6q23.3 | 24% | NHSL1 | protein coding | intronic | 3.0E-05 | 1 | 4 | ALL LEUKEMIA RELATED UNRELATED | sGvHD |
| rs7765573 | 6q23.3 | 26% | NHSL1 | protein coding | intronic | 6.8E-05 | 1 | 4 | ALL LEUKEMIA RELATED UNRELATED | sGvHD |
| rs76262569 | 6q25.1 | 8% | AKAP12 | protein coding | intronic | 1.3E-05 | 1 | 1 | LYMPHOMA | sGvHD |
| rs9383875 | 6q25.1 | 14% | AKAP12 | protein coding | intronic | 5.7E-05 | 1 | 1 | LYMPHOMA | sGvHD |
| rs9322363 | 6q25.1 | 48% | SYNE1 | protein coding | intronic | 8.4E-05 | 1 | 1 | LYMPHOMA | EFS |
| rs79730312 | 6q25.1 | 2% | SYNE1 | protein coding | intronic | 2.9E-05 | 1 | 4 | ALL LEUKEMIA RELATED UNRELATED | sGvHD |
| rs4146016 | 7p21.2 | 49% | ISPD | protein coding | intronic | 9.4E-05 | 1 | 4 | ALL RELATED UNRELATED LEUKEMIA | OS |
| rs4565368 | 7p21.2 | 32% | ISPD | protein coding | intronic | 6.3E-05 | 1 | 1 | REGENSBURG | OS |
| rs7787719 | 7q33 | 35% | (CNOT4,STRA8) | intergenic | intergenic | 7.6E-05 | 1 | 1 | REGENSBURG | OS |
| rs6960750 | 7q33 | 46% | (CNOT4,STRA8) | intergenic | intergenic | 3.8E-05 | 1 | 1 | REGENSBURG | OS |
| rs1047384 | 8p21.2 | 13% | SLC25A37 | protein coding | up/downstream | 9.0E-05 | 1 | 1 | LYMPHOMA | OS |
| rs13261 | 8p21.2 | 3% | SLC25A37 | protein coding | up/downstream | 2.1E-05 | 1 | 1 | LYMPHOMA | OS |
| rs7828768 | 8q12.1 | 31% | RP11-246K15.1 | lincRNA | intronic | 1.5E-06 | 1 | 1 | NEWCASTLE | OS |
| rs2720685 | 8q24.21 | 37% | PVT1 | processed transcript | intronic | 7.2E-05 | 2 | 4 | ALL LEUKEMIA RELATED UNRELATED | EFS |
| rs10505506 | 8q24.21 | 37% | PVT1 | processed transcript | intronic | 1.1E-05 | 2 | 4 | ALL LEUKEMIA RELATED UNRELATED | EFS |
| rs6982570 | 8q24.21 | 18% | PVT1 | processed transcript | intronic | 2.0E-05 | 2 | 4 | ALL LEUKEMIA RELATED UNRELATED | sGvHD |
| rs79230602 | 8q24.21 | 7% | PVT1 | processed transcript | intronic | 5.4E-05 | 1 | 1 | LYMPHOMA | EFS |
| rs7010574 | 8q24.21 | 18% | PVT1 | processed transcript | intronic | 5.4E-05 | 1 | 1 | LYMPHOMA | EFS |
| rs11142594 | 9q21.12 | 16% | TRPM3 | protein coding | intronic | 4.8E-05 | 1 | 1 | LYMPHOMA | OS |
| rs11789669 | 9q21.12 | 3% | TRPM3 | protein coding | intronic | 1.0E-05 | 1 | 5 | ALL UNRELATEDTED LEUKEMIA RELATED UNRELATED | OS |
| rs73539137 | 9q33.2 | 4% | TTLL11 | protein coding | intronic | 7.3E-06 | 1 | 1 | NEWCASTLE | EFS |
| rs7047948 | 9q33.2 | 7% | TTLL11 | protein coding | coding | 5.9E-06 | 1 | 1 | NEWCASTLE | EFS |
| rs10901875 | 10q26.13 | 23% | (RPS27P18,MRPS21P6) | intergenic | intergenic | 4.9E-05 | 1 | 1 | LYMPHOMA | EFS |
| rs7079662 | 10q26.13 | 24% | (RPS27P18,MRPS21P6) | intergenic | intergenic | 4.9E-05 | 1 | 1 | LYMPHOMA | EFS |
| rs1693656 | 10q26.13 | 31% | CTBP2 | protein coding | intronic | 3.4E-05 | 1 | 1 | LYMPHOMA | EFS |
| rs34822464 | 10q26.13 | 31% | CTBP2 | protein coding | intronic | 3.3E-05 | 1 | 1 | LYMPHOMA | EFS |
| rs12815656 | 12q24.13 | 14% | (RBM19,RP11-438N16.2) | intergenic | intergenic | 7.2E-05 | 1 | 1 | REGENSBURG | EFS |
| rs5744929 | 12q24.33 | 12% | POLE | protein coding | intronic | 8.1E-05 | 1 | 1 | REGENSBURG | EFS |
| rs5744944 | 12q24.33 | 39% | POLE | protein coding | up/downstream | 7.5E-05 | 1 | 1 | REGENSBURG | EFS |
| rs4941613 | 13q14.2 | 37% | RP11-279N8.1 | lincRNA | intronic | 7.0E-05 | 1 | 1 | NEWCASTLE | OS |
| rs4942703 | 13q14.2 | 47% | RP11-279N8.1 | lincRNA | intronic | 6.8E-05 | 1 | 1 | NEWCASTLE | OS |
| rs1903656 | 13q21.1 | 15% | (MIR5007,AL512655.1) | intergenic | intergenic | 8.9E-05 | 1 | 1 | REGENSBURG | EFS |
| rs17089861 | 13q21.1 | 9% | (MIR5007,AL512655.1) | intergenic | intergenic | 2.2E-06 | 1 | 1 | REGENSBURG | EFS |
| rs57713628 | 15q15.1 | 3% | GPR176 | protein coding | intronic | 6.4E-05 | 1 | 1 | NEWCASTLE | sGvHD |
| rs275715 | 15q15.1 | 10% | GPR176 | protein coding | intronic | 1.7E-06 | 1 | 1 | NEWCASTLE | sGvHD |
| rs72735848 | 15q21.1 | 4% | SEMA6D | protein coding | intronic | 4.4E-05 | 1 | 1 | NEWCASTLE | EFS |
| rs58813426 | 15q21.1 | 5% | SEMA6D | protein coding | intronic | 6.9E-06 | 1 | 1 | NEWCASTLE | EFS |
| rs7172348 | 15q22.2 | 22% | RP11-219B17.1 | protein coding | intronic | 4.7E-05 | 1 | 1 | NEWCASTLE | EFS |
| rs2113943 | 15q22.2 | 42% | RP11-219B17.1 | protein coding | intronic | 4.1E-05 | 1 | 1 | NEWCASTLE | EFS |
| rs8032013 | 15q25.1 | 25% | (CRABP1,WDR61) | intergenic | intergenic | 8.5E-05 | 1 | 1 | NEWCASTLE | sGvHD |
| rs882929 | 15q25.1 | 24% | (CRABP1,WDR61) | intergenic | intergenic | 6.8E-05 | 1 | 1 | NEWCASTLE | sGvHD |
| rs11631374 | 15q26.1 | 20% | SLCO3A1 | antisense | intronic | 3.2E-05 | 1 | 1 | REGENSBURG | EFS |
| rs1060205 | 15q26.1 | 16% | SLCO3A1 | antisense | intronic | 3.1E-05 | 1 | 1 | REGENSBURG | EFS |
| rs8067640 | 17p13.1 | 9% | WRAP53 | protein coding | intronic | 7.1E-05 | 1 | 1 | REGENSBURG | EFS |
| rs8079273 | 17p13.1 | 6% | WRAP53 | protein coding | intronic | 7.1E-05 | 1 | 1 | REGENSBURG | EFS |
| rs7405903 | 17q25.3 | 24% | B3GNTL1 | protein coding | intronic | 3.1E-05 | 1 | 1 | REGENSBURG | EFS |
| rs2168935 | 17q25.3 | 24% | B3GNTL1 | protein coding | intronic | 2.8E-05 | 1 | 1 | REGENSBURG | EFS |
| rs4797006 | 18p11.21 | 4% | MC5R | protein coding | up/downstream | 7.3E-05 | 1 | 4 | ALL LEUKEMIA RELATED UNRELATED | sGvHD |
| rs11080686 | 18p11.21 | 26% | MC5R | protein coding | up/downstream | 3.0E-05 | 1 | 4 | ALL LEUKEMIA RELATED UNRELATED | sGvHD |
| rs2868929 | 18p11.22 | 33% | (RP11-419J16.1,AP005271.1 | intergenic | intergenic | 5.1E-05 | 1 | 1 | NEWCASTLE | EFS |
| rs985785 | 18p11.22 | 35% | (RP11-419J16.1,AP005271.1 | intergenic | intergenic | 3.1E-05 | 1 | 1 | NEWCASTLE | EFS |
| rs567225 | 18q12.1 | 38% | (TRAPPC8,SLC25A52) | intergenic | intergenic | 6.0E-05 | 1 | 1 | LYMPHOMA | OS |
| rs676332 | 18q12.1 | 26% | (TRAPPC8,SLC25A52) | intergenic | intergenic | 3.1E-05 | 1 | 1 | LYMPHOMA | OS |
| rs73598219 | 20p12.3 | 13% | (RP4-764O22.1,snoR26) | intergenic | intergenic | 7.1E-05 | 1 | 1 | GOTTINGEN | sGvHD |
| rs73598217 | 20p12.3 | 9% | (RP4-764O22.1,snoR26) | intergenic | intergenic | 2.3E-05 | 1 | 1 | GOTTINGEN | sGvHD |
| rs4809591 | 20q13.12 | 18% | SLC13A3 | protein coding | up/downstream | 7.6E-06 | 1 | 1 | REGENSBURG | EFS |
| rs861025 | 20q13.12 | 21% | SLC13A3 | protein coding | intronic | 1.9E-06 | 1 | 1 | REGENSBURG | EFS |
| rs16996389 | 20q13.2 | 17% | (ZFP64,RN7SL603P) | intergenic | intergenic | 2.3E-05 | 1 | 1 | LYMPHOMA | sGvHD |
| rs16996391 | 20q13.2 | 14% | (ZFP64,RN7SL603P) | intergenic | intergenic | 2.3E-05 | 1 | 1 | LYMPHOMA | sGvHD |
| rs2092779 | 22q13.32 | 50% | (RPL35P8,WI2-81516E3.1) | intergenic | intergenic | 4.8E-05 | 1 | 1 | REGENSBURG | OS |
| rs12170462 | 22q13.32 | 22% | (RPL35P8,WI2-81516E3.1) | intergenic | intergenic | 2.1E-05 | 1 | 1 | REGENSBURG | OS |

* from patient genotypes
